# Factors associated with loneliness and social capital in a large volunteer sample of adults in England

**DOI:** 10.1186/s44263-026-00335-1

**Published:** 2026-10-02

**Authors:** Austen El-Osta, Mahmoud Al-Ammouri, Aos Alaa, Sami Altalib, Agustin Tristán-López, Azeem Majeed

**Affiliations:** 1 Self-Care Academic Research Unit, School of Public Health, Imperial College London, W12 0BZ London, UK; 2 Department of Primary Care & Public Health, School of Public Health, Imperial College London, W12 0BZ London, UK

**Keywords:** Loneliness, Social isolation, Social capital, Public health, Mental health, Social cohesion, Community interventions

## Abstract

**Background:**

Loneliness is a pressing public health concern with wide-ranging impacts on mental, physical and social wellbeing. Building on the Measuring Loneliness in England (INTERACT) Study which represents one of the largest volunteer-based studies of loneliness and social disconnection conducted in the United Kingdom (UK), we explore demographic, social and health-related factors associated with loneliness and social capital, using multiple validated measures.

**Methods:**

We analysed cross-sectional data from 135,725 community-dwelling adults across England. Loneliness was assessed using both the University of California, Los Angeles (UCLA) 3-item Loneliness Scale and the Office for National Statistics (ONS) Direct Measure of Loneliness (DMOL). Social capital was measured using a composite scale of neighbourhood trust, cohesion and reciprocity. Multivariable ordinal and binary logistic regression models were fitted to summarise mutually adjusted associations within the responding sample. The models were used as exploratory, sample-conditional descriptions and were not intended to estimate population parameters.

**Results:**

Younger age (particularly 16–25 years old), being single, unemployed or living with disability were consistently associated with higher loneliness across both scales. In contrast, respondents reporting nine or more friends had substantially lower odds of loneliness (UCLA: adjusted odds ratio (aOR) = 0.09, 95% model-based interval (MBI): 0.09–0.10; DMOL: aOR = 0.16, 95% MBI: 0.15–0.17). University education was associated with higher loneliness on the UCLA scale but lower loneliness on the DMOL. High social capital was more frequently reported among older, married and retired respondents. Respondents with long-term conditions or disability had reduced odds of high social capital (long-term conditions: aOR = 0.82, 95% MBI: 0.80–0.85; disability: aOR = 0.77, 95% MBI: 0.74–0.80).

**Conclusions:**

This study identified several sociodemographic, social and health-related factors associated with loneliness, although some associations varied according to the loneliness measure used. Because participants comprised a non-probability volunteer sample, the estimates should not be interpreted as population parameters, causal effects or confirmatory evidence. The findings are hypothesis-generating and require confirmation in representative, longitudinal and intervention-focused studies.

**Supplementary Information:**

The online version contains supplementary material available at https://doi.org/10.1186/s44263-026-00335-1.

## Background

Loneliness is increasingly recognised as a critical public health challenge, associated with profound implications for mental, physical and social wellbeing [1]. Accumulating evidence links loneliness to depression, anxiety, cardiovascular disease, cognitive decline and elevated mortality risk highlighting its significance as a biopsychosocial determinant of health [2]. While the COVID-19 pandemic intensified public awareness and policy interest, the epidemiology of loneliness remains underexplored, particularly in terms of its social determinants, distribution across diverse populations and contextual factors including social capital [3].

To address this evidence gap, the Measuring Loneliness in England (INTERACT) Study was launched as one of the largest volunteer-based investigations of loneliness and social disconnection conducted in the United Kingdom (UK), using the University of California, Los Angeles (UCLA) 3-item Loneliness Scale (UCLA-3) [4] and Office for National Statistics (ONS) Direct Measure of Loneliness (DMOL) [5]. The first paper to introduce the INTERACT study [6] reported descriptive findings from over 135,000 adults, describing substantial reported loneliness and unequal distribution within that sample. Young adults, ethnic minorities, urban residents and those living with disability or unemployment reported comparatively high loneliness within that sample. Older adults, especially those aged over 65 years, also emerged as a population of interest. Within the INTERACT sample, older respondents reported lower average loneliness scores than younger respondents, although frequent loneliness was also reported by some older participants. Within this group, widowed individuals, those living alone and people with long-term conditions or disabilities more frequently reported higher levels of social disconnection. Importantly, the descriptive findings highlighted stark variations in loneliness levels even among individuals with seemingly similar sociodemographic characteristics, suggesting the influence of underlying structural and contextual factors such as social capital.

This second paper builds on those foundational findings by conducting a large adjusted analysis of factors associated with loneliness, drawing on a sample of over 135,000 participants and a broad set of socio-demographic, social and health-related variables across two validated loneliness measures. It applies advanced statistical modelling to estimate adjusted associations between loneliness and key sociodemographic, socioeconomic and health-related characteristics across the life course without implying predictive performance or causal relationships. In doing so, it provides new insights into the complex sociodemographic, social and health-related factors associated with loneliness across different age groups, situating ageing within a broader multidimensional context.

A key focus of this study is social capital [3], measured through indicators of neighbourhood trust, perceived support and community cohesion. Social capital was analysed as a separate contextual outcome to examine how it varied across respondent characteristics. This contextual dimension is particularly relevant to older adults, who may face reduced mobility, bereavement and shrinking social networks [7].

Building on the descriptive architecture established in our previous work [6], this analysis shifts from pattern characterisation to estimation of conditional associations within the sample, enabling quantification of gradients and the identification of converging correlates across measures. This second paper makes three primary contributions. First, it provides adjusted association estimates for a broad set of loneliness correlates, including common variables such as household composition, caregiving roles and frequency of social contact. Second, it examines sociodemographic, social and health-related factors associated with high social capital as a complementary contextual outcome. Third, it explores variation in loneliness and social capital across respondent subgroups, considering contextual factors where measured. Crucially, this paper highlights age-related differences in loneliness across adult respondents and generates hypotheses about age-sensitive and life-stage-informed approaches to loneliness, including multigenerational public health strategies that acknowledge both the distinct and overlapping loneliness risks faced by younger and older people [7, 8]. The findings presented here generate hypotheses for the design and evaluation of targeted and equity-informed interventions, particularly those aimed at improving social connectedness and resilience in an ageing society.

The primary aim of this study was to estimate adjusted associations between loneliness and key demographic, socioeconomic, social and health-related factors within a large non-probability sample. Secondary aims were to (i) describe the distribution of loneliness and social capital across sociodemographic and health-related subgroups, (ii) examine associations between these factors and subjective loneliness using two validated measures, (iii) examine factors associated with social capital, and (iv) identify consistent patterns across models to inform hypothesis generation.

## Methods

This study is a cross-sectional secondary analysis of the Measuring Loneliness in England (INTERACT) online survey, using a non-probability volunteer sample of adults in England. Social connection is conceptualised as an overarching construct encompassing subjective loneliness, structural social contact (e.g., number of friends or relatives) and contextual dimensions captured through social capital. These components are analytically distinguished, with loneliness treated as a subjective outcome, social contact as a structural exposure, and social capital as a contextual construct.

### Study design and data source

The present analysis uses responses collected between 1 March and 31 July 2023 via Qualtrics [6]. Full methodological details, including study design, recruitment and data collection procedures, are provided in our previous work [6]. The survey is included in Supplementary Material 1.

### Participants and sampling

A total of 135,725 adults aged 16 years and older were recruited via National Health Service (NHS) primary care networks, voluntary sector organizations and the National Institute for Health and Care Research (NIHR) Be Part of Research Network. The recruitment strategy aimed at demographic and geographic diversity, with targeted outreach to underrepresented populations. Eligibility criteria and exclusion details have been described elsewhere [6].

### Measures

Loneliness was assessed indirectly using the validated UCLA Loneliness Scale, with response options categorized as never/hardly ever (scored as 1), some of the time (scored as 2) and often (scored as 3). Each question was scored from 1 to 3 and the total score ranged from 3 to 9. The total scores were further categorized into three levels of loneliness: no loneliness (score = 3), moderate loneliness (score = 4–6) and severe loneliness (score = 7–9). To facilitate direct comparison with the three-category DMOL outcome, the UCLA-3 summary score was collapsed into three ordered categories representing low (3), moderate (4–6) and high (7–9) loneliness. Although this harmonisation improved comparability between the two loneliness measures, collapsing categories inevitably reduced outcome granularity and may have attenuated variation within the UCLA-3 measure.

Given the absence of universally agreed cut-offs for the UCLA-3, scores were categorised pragmatically to reflect increasing severity while preserving ordinal structure for analysis. This pragmatic categorisation approach is consistent with prior population-based studies that have grouped UCLA-3 scores to reflect increasing severity while preserving ordinal structure for regression modelling [9]. A single-item DMOL recommended by the ONS was also included in our study. Crucially, the primary analyses retained the ordinal nature of the outcome, minimising reliance on arbitrary thresholds. Differences between UCLA-3 and DMOL estimates reflect established methodological differences between indirect multi-item and direct single-item measures, rather than inconsistency in findings.

To measure social capital, we used a seven-item Likert scale with four response categories, based on an instrument developed by Sampson et al. [10]. Response categories were dichotomised such that “agree” or “strongly agree” were scored as 1, and “disagree” or “strongly disagree” as 0, reflecting the presence versus absence of key neighbourhood attributes (e.g., trust, cohesion, shared values), consistent with prior applications of collective efficacy frameworks. Scores were summed to produce an overall index ranging from 0 to 7. Two negatively worded items (“People in this neighbourhood generally don’t get along with each other” and “People in this neighbourhood do not share the same values”) were reverse-coded. The summed index was subsequently dichotomised at the median (score = 4) to classify low (0–4) versus high (> 4) social capital, facilitating interpretation and binary logistic regression modelling of high versus low social capital. Dichotomisation was used to facilitate interpretability in regression models; however, we acknowledge this may reduce variability and should be interpreted as a pragmatic modelling decision rather than a theoretically derived threshold. While this approach may reduce granularity, it supports comparability with existing literature [11].

All scores for loneliness and social capital in this analysis were derived using a classical test theory approach by summing item responses. Rasch model-derived person measures in logits are reported separately in our published psychometric evaluation [12]. Internal consistency of the UCLA-3 and the seven-item social-capital measure was assessed using Cronbach’s α and McDonald’s ω. Because the DMOL is a single-item measure, internal-consistency reliability was not applicable. The UCLA-3 had Cronbach’s α = 0.897 and McDonald’s ω = 0.947; the seven-item social-capital measure had Cronbach’s α = 0.847 and McDonald’s ω = 0.887. Reliability after binary item scoring was assessed using the Kuder–Richardson Formula 20 (KR-20 = 0.791).

In addition to loneliness and social capital, a range of socio-demographic, social and health-related variables were included as covariates selected a priori based on established theoretical frameworks (see Statistical Analysis). These comprised age group, gender, ethnicity, educational attainment, employment status and marital status, as well as indicators of social connectedness (number of friends and relatives, household size, presence of children and pet ownership) and health status (self-reported disability and long-term conditions). All variables were self-reported and categorised as described in the Statistical Analysis section and corresponding tables. Complete item wording, response options and scoring procedures are provided in Supplementary material 2: Tables S1–S3. Descriptive distributions are provided in Supplementary material 3: Tables S4–S6.

### Handling of missing data

We assessed the extent of missing data across all variables in the dataset. Overall, 6.8% of the dataset values were missing. Prior to imputation, we used Little’s missing completely at random (MCAR) test to examine the missing data mechanism, which suggested that the data were not missing completely at random. Therefore, we proceeded with multiple imputations using the Multivariate Imputation by Chained Equations (MICE) algorithm, implemented via the ‘*mice*’ package in R for comparison and robustness checking with complete case analysis. The missing data pattern was inspected using the *md.pattern() function* and methods were tailored based on variable types: polytomous regression (*polyreg*) for nominal categorical variables (e.g., gender, ethnicity), proportional odds models (*polr*) for ordinal variables (e.g., age group, number of relatives and friends, household size) and logistic regression (*logreg*) for binary variables (e.g., having children, pet ownership). We generated five imputed datasets (m = 5) with 40 iterations per dataset (maxit = 40), setting a random seed (123) for reproducibility. Diagnostic plots confirmed algorithm convergence, showing stable mean and standard deviation trends across iterations. Post-imputation, analyses were conducted on each dataset individually and the results were pooled using Rubin’s rules to account for imputation uncertainty (Supplementary material 4: Figs S1–S5 and Tables S7–S9). The primary analyses presented in this paper are based on complete-case models, with multiply imputed analyses conducted as sensitivity analyses to assess robustness.

### Statistical analysis

Covariates were selected a priori based on established theoretical frameworks, including the Social Determinants of Health [13] and biopsychosocial models of social isolation [14]. These frameworks informed the inclusion of structural (e.g., education, employment), demographic (e.g., age, gender, ethnicity), relational (e.g., marital status, number of friends and relatives, household composition), behavioural/contextual (e.g., pet ownership, caregiving roles) and health-related factors (e.g., disability, long-term conditions).

Participant characteristics were summarized using frequencies and percentages. Because participants were recruited through non-probability volunteer sampling and individual inclusion probabilities were unknown, no estimates of population prevalence or design-based sampling error were calculated.

Multivariable cumulative-logit proportional-odds models were fitted for the UCLA-3 and DMOL outcomes, and binary logistic regression was fitted for the dichotomised social-capital outcome. For each three-category loneliness outcome, the models estimated the cumulative odds of moderate or severe versus low/no loneliness, and severe versus low/no or moderate loneliness. Separate threshold-specific intercepts were estimated, while covariate coefficients were constrained to be constant across thresholds under the proportional-odds assumption. No single outcome category therefore served as the reference category. Outcomes were ordered from lower to higher loneliness, such that adjusted odds ratios greater than 1 indicate higher cumulative odds of membership in a more severe loneliness category.

These models were used as exploratory descriptive tools to summarise mutually adjusted associations within the responding sample. They were not intended to estimate population parameters, test population hypotheses, or establish causal or predictive relationships. No survey weighting, raking, or post-stratification procedures were applied; accordingly, all analyses describe the responding sample and should not be interpreted as population-representative estimates.

Analyses were performed using all available complete cases for each outcome separately. A single complete-case dataset across all outcomes was not used because this would have unnecessarily excluded respondents with complete information for one outcome but missing data for another. Had a single complete-case dataset been imposed across all three outcomes and model covariates, the available sample would have been 117,781.

Results are reported as adjusted odds ratios (aORs) with 95% model-based intervals (MBIs). These intervals quantify uncertainty under the fitted regression model, conditional on the analysed respondents, observed covariates, and model assumptions. They do not incorporate uncertainty arising from volunteer recruitment, unknown inclusion probabilities, non-coverage, or self-selection and should not be interpreted as population confidence intervals. No conclusions were based on null-hypothesis significance testing. All statistical analyses were performed using R software version 4.2.2.

### Reporting guidelines

The study was reported in accordance with the Strengthening the Reporting of Observational Studies in Epidemiology checklist and the Checklist for Reporting Results of Internet E-Surveys (CHERRIES) [15, 16]. The completed reporting checklists are provided in Supplementary material 5: Table S10 and Supplementary material 6: Table S11, respectively.

## Results

Results are presented as descriptive distributions and adjusted within-sample model summaries. All percentages describe the responding sample and should not be interpreted as population prevalence estimates. The adjusted models summarise conditional associations among analysed respondents and do not support population-level, causal or predictive inference. Descriptive distributions of social capital were based on 120,583 respondents with available social capital data, whereas the adjusted regression model included 117,781 complete observations across all covariates. All regression results presented are based on complete-case analysis, with imputed models yielding consistent estimates (Supplementary material 4: Tables S7–S9).

### Observed distribution of loneliness and social capital

Among 135,722 respondents with complete loneliness-outcome data, loneliness varied across demographic, social and health-related groups. Supplementary material 3: Table S4 presents the observed proportions of low/no, moderate and severe loneliness according to the UCLA-3 across key demographic and social subgroups. Overall, 30.5% of respondents were classified as experiencing severe loneliness on the UCLA-3, whereas 16.5% reported feeling lonely “often or always” on the DMOL. Within the INTERACT sample, severe UCLA-3 loneliness was most frequently observed among younger adults and decreased progressively with age, from 54.9% among respondents aged 16–25 years to 16.4% among those aged over 65 years. Conversely, the proportion classified as having low or no UCLA-3 loneliness increased from 7.0% among respondents aged 16–25 years to 42.9% among those aged over 65 years.

Patterns of loneliness measured using DMOL broadly complemented those observed with the UCLA-3 and provided a direct single-item measure of loneliness frequency. Supplementary material 3: Table S5 summarises the observed proportions of low (never/hardly ever), moderate (occasionally/some of the time) and severe (often/always) loneliness across key demographic and social variables. Within the INTERACT sample, 47.5% of respondents aged ≥ 65 years reported “never or hardly ever” feeling lonely on the DMOL measure. Females consistently reported higher levels of moderate and severe loneliness than males across both scales. Notably, respondents identifying as ‘Other’ gender had a high within-group proportion reporting severe loneliness. Loneliness was also socially patterned by relationship status, employment and health. Single and divorced respondents generally reported higher loneliness, whereas married or civil-partnered respondents reported lower loneliness; patterns among widowed respondents varied by measure and category. Unemployed respondents, respondents with disabilities and those with long-term conditions more frequently reported moderate or severe loneliness across both UCLA and DMOL measures.

The observed distributions of low and high social capital across demographic and social characteristics are presented in Supplementary material 3: Table S6. Social capital distributions were based on 120,583 respondents with complete seven-item social-capital scores.

The adjusted social-capital model included 117,781 respondents with complete outcome and covariate data. Social capital was measured using a composite scale of neighbourhood trust, cohesion and support. Higher social capital was more frequently observed among older adults, married individuals and those with a greater number of friends or relatives. Conversely, lower social capital was more frequently observed among younger respondents, individuals living alone, the unemployed and those in poor health.

Adjusted analyses were conducted to summarise mutually adjusted associations between loneliness and a range of sociodemographic, health and social variables within the responding sample. Ordinal logistic regression models were fitted for UCLA-3 and DMOL, and binary logistic regression was fitted for social capital. These models are presented as exploratory, sample-conditional descriptions rather than tests of population hypotheses.

Multivariable logistic regression was used to estimate mutually adjusted associations between the included sociodemographic, health and social factors and the loneliness and social-capital outcomes. Ordinal models were applied to UCLA and DMOL scores and binary models to social capital. All models adjusted for age, gender, ethnicity, education, employment, marital status, social contacts and health status. This segment presents the findings from multivariable models for UCLA, DMOL and social capital.

### Ordinal regression analysis: UCLA Loneliness Scale

**Fig. 1:**
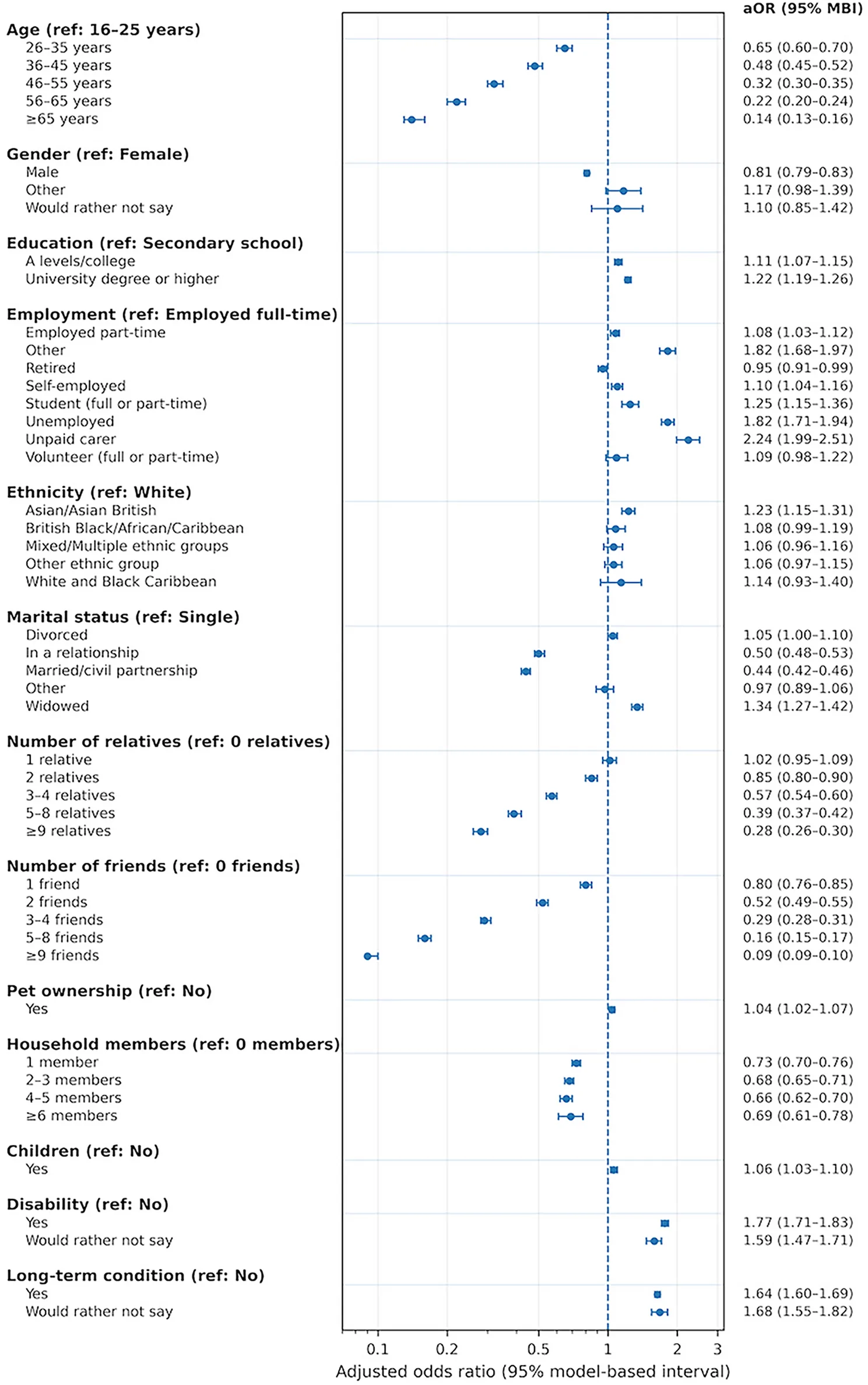
Forest plot showing adjusted odds ratios and 95% model-based intervals (MBI) from ordinal logistic regression for socio-demographic, social and health-related factors associated with loneliness measured using UCLA-3; *N* = 122,257

Multivariable ordinal logistic regression modelling identified several factors associated with greater UCLA-3 loneliness after adjustment. The analysis of unadjusted and adjusted odds ratios (OR) for various demographic and social factors associated with UCLA-3 loneliness are shown in Table 1; Fig. 1.

**Table 1:** Association between demographic and social factors and loneliness as measured by UCLA Loneliness Scale; *N* = 122,257 observations in adjusted model

Age	Unadjusted OR (95% MBI)	aOR (95% MBI)
16–25	Ref.	Ref.
26–35	0.70 (0.67–0.74)	0.65 (0.60–0.70)
36–45	0.59 (0.56–0.62)	0.48 (0.45–0.52)
46–55	0.43 (0.41–0.45)	0.32 (0.30–0.35)
56–65	0.26 (0.25–0.28)	0.22 (0.20–0.24)
> 65	0.15 (0.15–0.16)	0.14 (0.13–0.16)
**Gender**		
Female	Ref.	Ref.
Male	0.79 (0.77–0.80)	0.81 (0.79–0.83)
Other	3.37 (2.93–3.87)	1.17 (0.98–1.39)
Would rather not say	1.98 (1.68–2.34)	1.10 (0.85–1.42)
**Education**		
Secondary school	Ref.	Ref.
A levels/College	1.02 (1.00–1.05)	1.11 (1.07–1.15)
University Degree or higher	0.80 (0.77–0.82)	1.22 (1.19–1.26)
**Employment**		
Employed full-time	Ref.	Ref.
Employed part-time	0.79 (0.77–0.83)	1.08 (1.03–1.12)
Other	3.35 (3.13–3.57)	1.82 (1.68–1.97)
Retired	0.42 (0.41–0.43)	0.95 (0.91–0.99)
Self-employed	0.68 (0.65–0.72)	1.10 (1.04–1.16)
Student (full or part-time)	2.28 (2.14–2.43)	1.25 (1.15–1.36)
Unemployed	4.71 (4.47–4.96)	1.82 (1.71–1.94)
Unpaid carer	2.64 (2.39–2.92)	2.24 (1.99–2.51)
Volunteer (full or part-time)	0.60 (0.54–0.66)	1.09 (0.98–1.22)
**Ethnicity**		
White	Ref.	Ref.
Asian/Asian British	1.79 (1.70–1.88)	1.23 (1.15–1.31)
British Black/African/Caribbean	1.74 (1.61–1.88)	1.08 (0.99–1.19)
Mixed/Multiple ethnic groups	1.85 (1.70–2.01)	1.06 (0.96–1.16)
Other ethnic group	1.60 (1.49–1.72)	1.06 (0.97–1.15)
White and Black Caribbean	2.33 (1.96–2.77)	1.14 (0.93–1.40)
**Marital Status**		
Single	Ref.	Ref.
Divorced	0.62 (0.59–0.64)	1.05 (1.00–1.10)
In a relationship	0.41 (0.39–0.43)	0.50 (0.48–0.53)
Married / Civil partnership	0.18 (0.17–0.18)	0.44 (0.42–0.46)
Other	0.76 (0.70–0.81)	0.97 (0.89–1.06)
Widowed	0.45 (0.43–0.47)	1.34 (1.27–1.42)
**Number of relatives**		
0 relatives	Ref.	Ref.
1 relative	0.92 (0.87–0.98)	1.02 (0.95–1.09)
2 relatives	0.72 (0.68–0.75)	0.85 (0.80–0.90)
3 or 4 relatives	0.37 (0.35–0.39)	0.57 (0.54–0.60)
5–8 relatives	0.16 (0.16–0.17)	0.39 (0.37–0.42)
9 or more relatives	0.09 (0.08–0.09)	0.28 (0.26–0.30)
**Number of friends**		
0 friends	Ref.	Ref.
1 friend	0.80 (0.76–0.84)	0.80 (0.76–0.85)
2 friends	0.47 (0.45–0.49)	0.52 (0.49–0.55)
3 or 4 friends	0.22 (0.21–0.23)	0.29 (0.28–0.31)
5–8 friends	0.10 (0.10–0.11)	0.16 (0.15–0.17)
9 or more friends	0.05 (0.05–0.06)	0.09 (0.09–0.10)
**Having pet**		
No	Ref.	Ref.
Yes	1.24 (1.22–1.27)	1.04 (1.02–1.07)
**Number of household members**		
0 members	Ref.	Ref.
1 member	0.37 (0.36–0.38)	0.73 (0.70–0.76)
2–3 members	0.62 (0.60–0.64)	0.68 (0.65–0.71)
4–5 members	0.72 (0.68–0.75)	0.66 (0.62–0.70)
> 5 members	1.08 (0.98–1.19)	0.69 (0.61–0.78)
**Having children**		
No	Ref.	Ref.
Yes	0.48 (0.47–0.49)	1.06 (1.03–1.10)
**Having disability**		
No	Ref.	Ref.
Yes	3.51 (3.42–3.61)	1.77 (1.71–1.83)
Would rather not say	3.49 (3.28–3.73)	1.59 (1.47–1.71)
**Having long-term condition**		
No	Ref.	Ref.
Yes	2.76 (2.70–2.82)	1.64 (1.60–1.69)
Would rather not say	3.05 (2.85–3.27)	1.68 (1.55–1.82)

Note: *Adjusted models included age*,* gender*,* ethnicity*,* education*,* employment*,* marital status*,* social contacts*,* household size*,* pet ownership*,* children*,* disability and long-term conditions. Model-based intervals are conditional on the fitted model and analysed respondents; they do not account for uncertainty or bias arising from non-probability selection and do not support population-level inference. Abbreviations: OR*,* odds ratio; aOR*,* adjusted odds ratio; MBI*,* model-based interval; N*,* number of observations; Ref.*,* reference category; UCLA*,* University of California*,* Los Angeles; UCLA-3*,* three-item UCLA Loneliness Scale*

The adjusted odds ratios (aORs) reported here reflect associations after simultaneous adjustment for all included covariates and should be interpreted as conditional estimates rather than causal effects. Age showed the strongest inverse association with loneliness across models. Compared with individuals aged 16–25 years (reference group), older participants reported substantially lower odds of higher loneliness scores. Relative to respondents aged 16–25 years, those aged 26–35 years had 35% lower adjusted odds of being in a more severe loneliness category (aOR = 0.65, 95% MBI: 0.60–0.70), while those aged ≥ 65 years had 86% lower adjusted odds (aOR = 0.14, 95% MBI: 0.13–0.16). Gender showed an adjusted association with loneliness within the responding sample. Males had lower adjusted odds of being in a more severe loneliness category relative to females (aOR = 0.81, 95% MBI: 0.79–0.83). While the ‘Other’ gender category showed elevated unadjusted loneliness, the adjusted estimate was close to the null and its model-based interval included 1.00 (aOR = 1.17, 95% MBI: 0.98–1.39).

Educational attainment showed a heterogeneous association with loneliness across measures. Relative to respondents with secondary-school education, university graduates had higher adjusted odds of being in a more severe loneliness category in the UCLA-3 model (aOR = 1.22, 95% MBI: 1.19–1.26). Relative to respondents in full-time employment, unemployed respondents had higher adjusted odds (aOR = 1.82, 95% MBI: 1.71–1.94), as did unpaid carers (aOR = 2.24, 95% MBI: 1.99–2.51), while retired respondents had slightly lower adjusted odds (aOR = 0.95, 95% MBI: 0.91–0.99).

Marital status showed some of the strongest adjusted social associations. Relative to single respondents, those who were married or in a civil partnership had markedly lower adjusted odds of being in a more severe loneliness category (aOR = 0.44, 95% MBI: 0.42–0.46), as did those who were in a relationship (aOR = 0.50, 95% MBI: 0.48–0.53). Widowed respondents had higher adjusted odds relative to single respondents (aOR = 1.34, 95% MBI: 1.27–1.42). Social contact showed a graded inverse association. Relative to respondents reporting no friends, those reporting nine or more friends had substantially lower adjusted odds of more severe loneliness (aOR = 0.09, 95% MBI: 0.09–0.10). Relative to respondents reporting no relatives, those reporting nine or more relatives had substantially lower adjusted odds of being in a more severe UCLA-3 loneliness category (aOR = 0.28, 95% MBI: 0.26–0.30).

Health-related factors were also strongly associated with loneliness. Relative to respondents reporting no disability, respondents with a disability had 77% higher adjusted odds of being in a more severe loneliness category (aOR = 1.77, 95% MBI: 1.71–1.83). Relative to respondents reporting no long-term condition, those with a long-term condition had 64% higher adjusted odds (aOR = 1.64, 95% MBI: 1.60–1.69).

Models adjust for age, gender, ethnicity, education, employment, marital status, social contacts, household size, pet ownership, children, disability and long-term conditions. Reference categories are shown in Table 1. Values < 1 indicate lower odds of higher loneliness.

### Ordinal regression analysis: DMOL

**Fig. 2:**
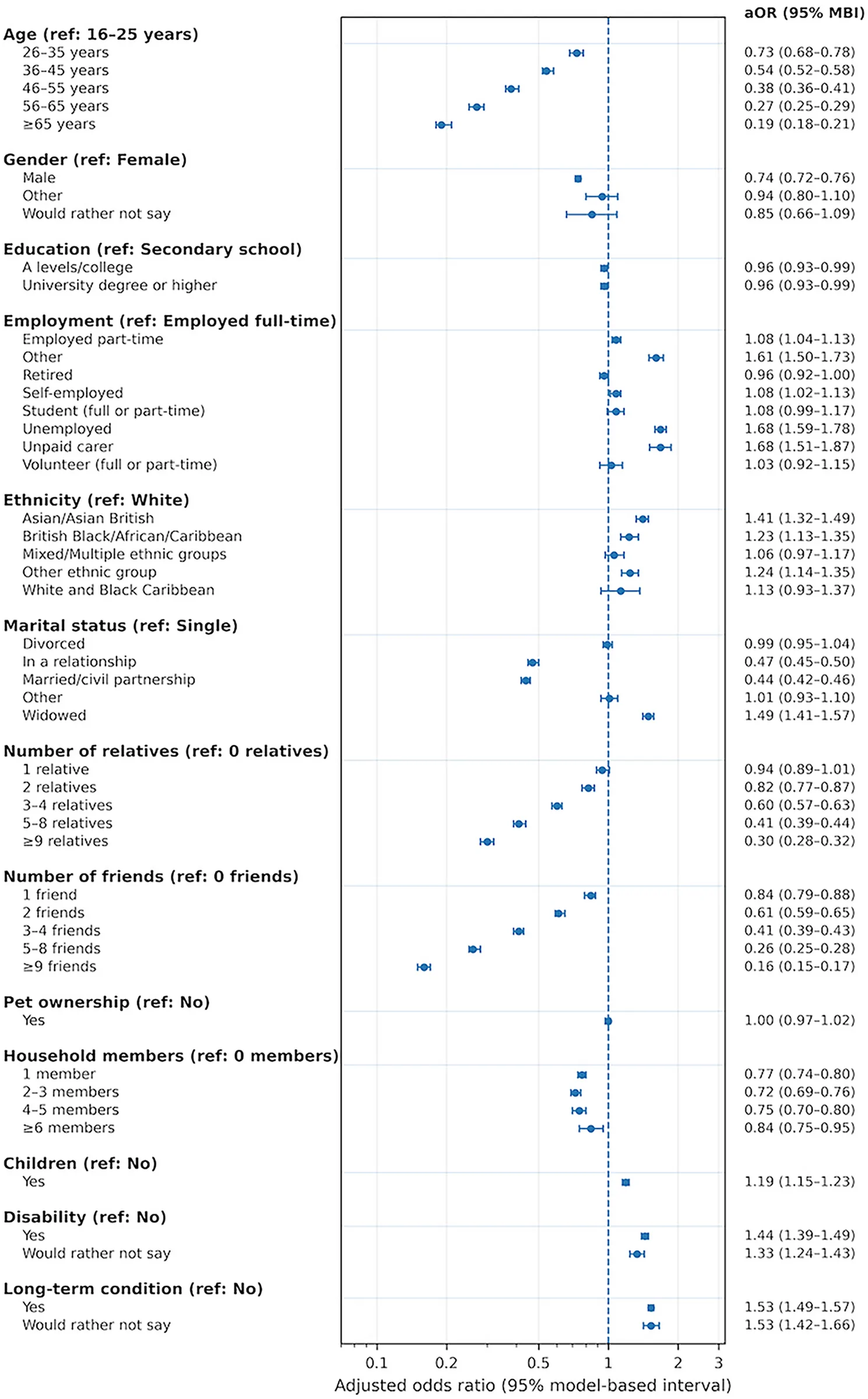
Forest plot showing adjusted odds ratios and 95% model-based intervals (MBI) from ordinal logistic regression for socio-demographic, social and health-related factors associated with loneliness using DMOL; *N* = 122,257

The analysis of unadjusted and adjusted odds ratios for various demographic and social factors associated with DMOL are shown in Table 2; Fig. 2. Findings from the DMOL model were directionally consistent with those from the UCLA-3 model, although the magnitudes of some associations differed. Age remained strongly associated with loneliness: respondents aged ≥ 65 had lower within-sample odds of frequent loneliness than respondents aged 16–25 years (aOR = 0.19, 95% MBI: 0.18–0.21). Males also had lower adjusted odds than females (aOR = 0.74, 95% MBI: 0.72–0.76). The adjusted estimate for respondents identifying as ‘Other’ gender was close to the null, and its model-based interval included 1.00 (aOR = 0.94, 95% MBI: 0.80–1.10).

**Table 2:** Association between demographic and social factors and loneliness as measured by DMOL; *N* = 122,257 observations in adjusted model

Age	Unadjusted OR (95% MBI)	aOR (95% MBI)
16–25	Ref.	Ref.
26–35	0.76 (0.72–0.80)	0.73 (0.68–0.78)
36–45	0.64 (0.61–0.67)	0.54 (0.52–0.58)
46–55	0.49 (0.46–0.51)	0.38 (0.36–0.41)
56–65	0.31 (0.29–0.32)	0.27 (0.25–0.29)
> 65	0.20 (0.19–0.21)	0.19 (0.18–0.21)
**Gender**		
Female	Ref.	Ref.
Male	0.73 (0.72–0.75)	0.74 (0.72–0.76)
Other	2.42 (2.12–2.76)	0.94 (0.80–1.10)
Would rather not say	1.73 (1.46–2.04)	0.85 (0.66–1.09)
**Education**		
Secondary school	Ref.	Ref.
A levels/College	0.93 (0.91–0.96)	0.96 (0.93–0.99)
University Degree or higher	0.70 (0.68–0.72)	0.96 (0.93–0.99)
**Employment**		
Employed full-time	Ref.	Ref.
Employed part-time	0.87 (0.84–0.90)	1.08 (1.04–1.13)
Other	2.96 (2.78–3.15)	1.61 (1.50–1.73)
Retired	0.49 (0.47–0.50)	0.96 (0.92–1.00)
Self-employed	0.71 (0.68–0.75)	1.08 (1.02–1.13)
Student (full or part-time)	2.02 (1.89–2.15)	1.08 (0.99–1.17)
Unemployed	4.15 (3.96–4.35)	1.68 (1.59–1.78)
Unpaid carer	2.18 (1.98–2.40)	1.68 (1.51–1.87)
Volunteer (full or part-time)	0.63 (0.57–0.70)	1.03 (0.92–1.15)
**Ethnicity**		
White	Ref.	Ref.
Asian/Asian British	1.88 (1.78–1.98)	1.41 (1.32–1.49)
British Black/African/Caribbean	1.83 (1.70–1.98)	1.23 (1.13–1.35)
Mixed/Multiple ethnic groups	1.71 (1.58–1.86)	1.06 (0.97–1.17)
Other ethnic group	1.73 (1.60–1.86)	1.24 (1.14–1.35)
White and Black Caribbean	2.24 (1.88–2.67)	1.13 (0.93–1.37)
**Marital Status**		
Single	Ref.	Ref.
Divorced	0.65 (0.63–0.68)	0.99 (0.95–1.04)
In a relationship	0.39 (0.37–0.41)	0.47 (0.45–0.50)
Married / Civil partnership	0.20 (0.19–0.21)	0.44 (0.42–0.46)
Other	0.82 (0.76–0.88)	1.01 (0.93–1.10)
Widowed	0.58 (0.55–0.61)	1.49 (1.41–1.57)
**Number of relatives**		
0 relatives	Ref.	Ref.
1 relative	0.88 (0.83–0.93)	0.94 (0.89–1.01)
2 relatives	0.71 (0.67–0.74)	0.82 (0.77–0.87)
3 or 4 relatives	0.40 (0.38–0.42)	0.60 (0.57–0.63)
5–8 relatives	0.19 (0.18–0.20)	0.41 (0.39–0.44)
9 or more relatives	0.11 (0.10–0.12)	0.30 (0.28–0.32)
**Number of friends**		
0 friends	Ref.	Ref.
1 friend	0.82 (0.78–0.86)	0.84 (0.79–0.88)
2 friends	0.53 (0.50–0.55)	0.61 (0.59–0.65)
3 or 4 friends	0.29 (0.27–0.30)	0.41 (0.39–0.43)
5–8 friends	0.15 (0.15–0.16)	0.26 (0.25–0.28)
9 or more friends	0.08 (0.08–0.08)	0.16 (0.15–0.17)
**Having pet**		
No	Ref.	Ref.
Yes	1.16 (1.13–1.18)	1.00 (0.97–1.02)
**Number of household members**		
0 members	Ref.	Ref.
1 member	0.37 (0.36–0.38)	0.77 (0.74–0.80)
2–3 members	0.60 (0.59–0.62)	0.72 (0.69–0.76)
4–5 members	0.72 (0.69–0.76)	0.75 (0.70–0.80)
> 5 members	1.11 (1.01–1.23)	0.84 (0.75–0.95)
**Having children**		
No	Ref.	Ref.
Yes	0.56 (0.55–0.57)	1.19 (1.15–1.23)
**Having disability**		
No	Ref.	Ref.
Yes	2.89 (2.81–2.97)	1.44 (1.39–1.49)
Would rather not say	2.98 (2.78–3.17)	1.33 (1.24–1.43)
**Having long-term condition**		
No	Ref.	Ref.
Yes	2.43 (2.38–2.49)	1.53 (1.49–1.57)
Would rather not say	2.73 (2.55–2.92)	1.53 (1.42–1.66)

Note: *Adjusted models included age*,* gender*,* ethnicity*,* education*,* employment*,* marital status*,* social contacts*,* household size*,* pet ownership*,* children*,* disability and long-term conditions. Model-based intervals are conditional on the fitted model and analysed respondents; they do not account for uncertainty or bias arising from non-probability selection and do not support population-level inference. Abbreviations: OR*,* odds ratio; aOR*,* adjusted odds ratio; MBI*,* model-based interval; N*,* number of observations; Ref.*,* reference category; DMOL*,* Direct Measure of Loneliness*

Educational attainment showed different patterns across the two loneliness measures. Relative to respondents with secondary-school education, respondents with a university degree had slightly lower adjusted odds of being in a more severe DMOL loneliness category (aOR = 0.96, 95% MBI: 0.93–0.99), whereas the direction of association differed in the UCLA-3 model. Relative to respondents in full-time employment, unemployed respondents had higher adjusted odds of being in a more severe DMOL loneliness category (aOR = 1.68, 95% MBI: 1.59–1.78), as did unpaid carers (aOR = 1.68, 95% MBI: 1.51–1.87). Relative to single respondents, widowed respondents had higher adjusted odds (aOR = 1.49, 95% MBI: 1.41–1.57), whereas respondents who were married or in a civil partnership had lower adjusted odds (aOR = 0.44, 95% MBI: 0.42–0.46). Relative to respondents reporting no friends, those reporting nine or more friends had 84% lower adjusted odds of more severe loneliness (aOR = 0.16, 95% MBI 0.15–0.17).

Health variables mirrored previous findings. Relative to respondents reporting no disability, respondents with a disability had higher adjusted odds of being in a more severe DMOL loneliness category (aOR = 1.44, 95% MBI: 1.39–1.49). Relative to respondents reporting no long-term condition, those with a long-term condition also had higher adjusted odds (aOR = 1.53, 95% MBI: 1.49–1.57). Respondents with children had slightly higher adjusted odds relative to those without children (aOR = 1.19, 95% MBI: 1.15–1.23), while the estimate for pet ownership relative to no pet ownership was close to the null (aOR = 1.00, 95% MBI: 0.97–1.02).

Models adjust for age, gender, ethnicity, education, employment, marital status, social contacts, household size, pet ownership, children, disability and long-term conditions. Reference categories are shown in Table 2. Values < 1 indicate lower odds of higher loneliness.

### Binary regression analysis: social capital

**Fig. 3:**
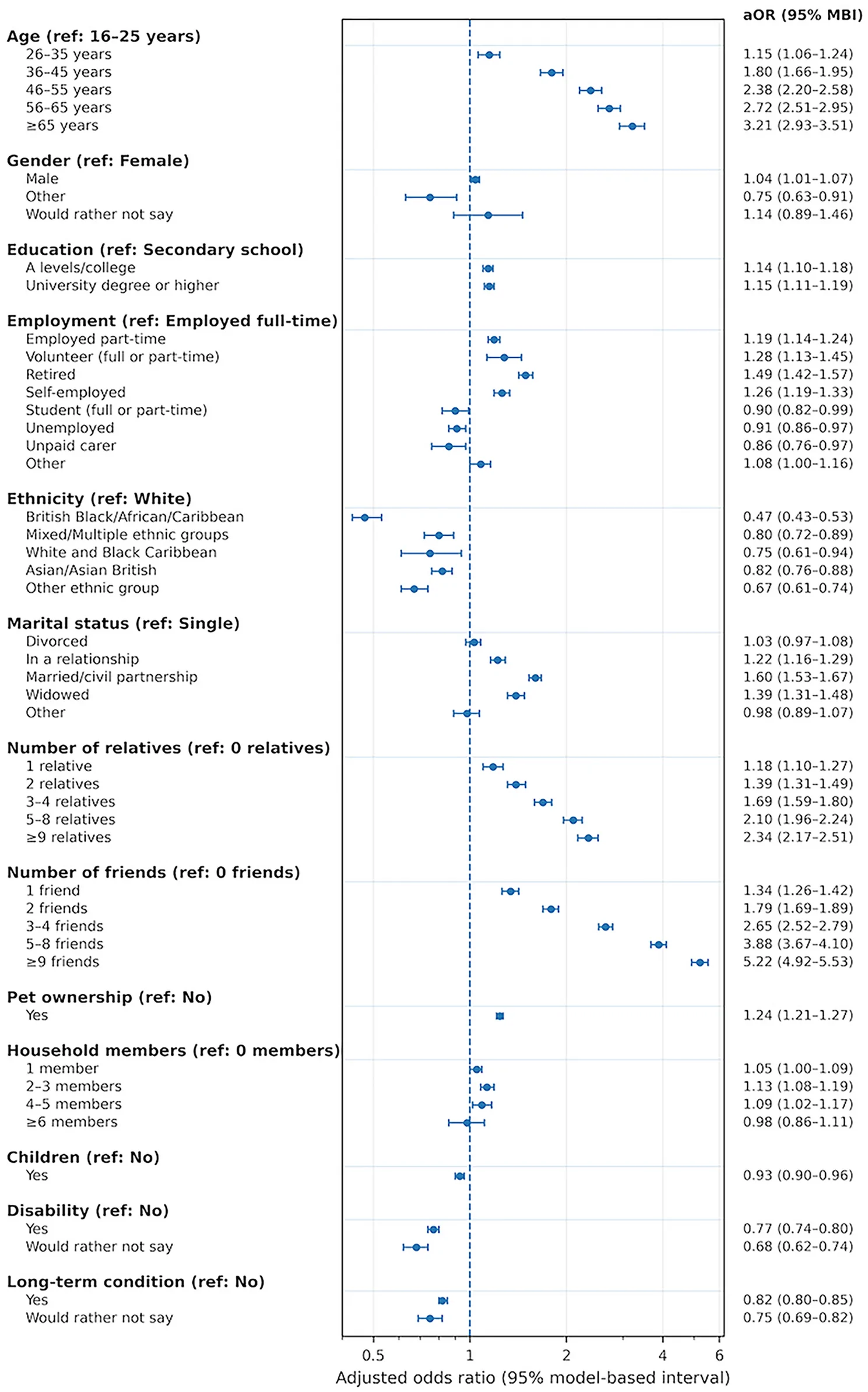
Forest plot showing adjusted odds ratios and 95% model-based intervals (MBI) from binary logistic regression for socio-demographic, social and health-related factors associated with social capital; *N* = 117,781

Table 3 presents adjusted odds ratios for various demographic and social factors associated with the Social Capital Scale; the corresponding forest plot is presented in Fig. 3. Binary logistic regression identified several factors associated with higher versus lower social capital. Age showed a strong positive association with high social capital. Relative to respondents aged 16–25 years, those aged 46–55 years had more than twice the adjusted odds of high social capital (aOR = 2.38, 95% MBI: 2.20–2.58), while those aged ≥ 65 years had over threefold higher adjusted odds (aOR = 3.21, 95% MBI: 2.93–3.51). Relative to single respondents, those who were married or in a civil partnership had higher adjusted odds of high social capital (aOR = 1.60, 95% MBI: 1.53–1.67), as did widowed respondents (aOR = 1.39, 95% MBI: 1.31–1.48). Relative to respondents in full-time employment, retired respondents had higher adjusted odds (aOR = 1.49, 95% MBI: 1.42–1.57), whereas unemployed respondents (aOR = 0.91, 95% MBI: 0.86–0.97) and unpaid carers (aOR = 0.86, 95% MBI: 0.76–0.97) had lower adjusted odds.

**Table 3:** Association between demographic and social factors and social capital scale; *N* = 117,781 observations in adjusted model

Age	Unadjusted OR (95% MBI)	aOR (95% MBI)
16–25	Ref.	Ref.
26–35	1.19 (1.11–1.27)	1.15 (1.06–1.24)
36–45	1.79 (1.68–1.90)	1.80 (1.66–1.95)
46–55	2.38 (2.25–2.53)	2.38 (2.20–2.58)
56–65	3.33 (3.14–3.52)	2.72 (2.51–2.95)
> 65	5.37 (5.08–5.68)	3.21 (2.93–3.51)
**Gender**		
Female	Ref.	Ref.
Male	1.04 (1.02–1.07)	1.04 (1.01–1.07)
Other	0.31 (0.26–0.37)	0.75 (0.63–0.91)
Would rather not say	0.56 (0.45–0.70)	1.14 (0.89–1.46)
**Education**		
Secondary school	Ref.	Ref.
A levels/College	1.09 (1.05–1.12)	1.14 (1.10–1.18)
University Degree or higher	1.30 (1.26–1.34)	1.15 (1.11–1.19)
**Employment**		
Employed full-time	Ref.	Ref.
Employed part-time	1.44 (1.38–1.49)	1.19 (1.14–1.24)
Volunteer (full or part-time)	1.93 (1.73–2.15)	1.28 (1.13–1.45)
Retired	2.54 (2.47–2.61)	1.49 (1.42–1.57)
Self-employed	1.72 (1.63–1.81)	1.26 (1.19–1.33)
Student (full or part-time)	0.53 (0.49–0.57)	0.90 (0.82–0.99)
Unemployed	0.42 (0.39–0.44)	0.91 (0.86–0.97)
Unpaid carer	0.67 (0.60–0.75)	0.86 (0.76–0.97)
Other	0.65 (0.61–0.70)	1.08 (1.00–1.16)
**Ethnicity**		
White	Ref.	Ref.
British Black/African/Caribbean	0.32 (0.29–0.35)	0.47 (0.43–0.53)
Mixed/Multiple ethnic groups	0.51 (0.46–0.56)	0.80 (0.72–0.89)
White and Black Caribbean	0.44 (0.36–0.53)	0.75 (0.61–0.94)
Asian/Asian British	0.54 (0.51–0.57)	0.82 (0.76–0.88)
Other ethnic group	0.51 (0.47–0.55)	0.67 (0.61–0.74)
**Marital status**		
single	Ref.	Ref.
Divorced	1.65 (1.58–1.73)	1.03 (0.97–1.08)
In a relationship	1.54 (1.47–1.60)	1.22 (1.16–1.29)
Married / Civil partnership	3.31 (3.21–3.41)	1.60 (1.53–1.67)
Widowed	3.20 (3.05–3.37)	1.39 (1.31–1.48)
Other	1.26 (1.17–1.37)	0.98 (0.89–1.07)
**Number of relatives**		
0 relatives	Ref.	Ref.
1 relative	1.25 (1.17–1.33)	1.18 (1.10–1.27)
2 relatives	1.55 (1.46–1.65)	1.39 (1.31–1.49)
3 or 4 relatives	2.32 (2.20–2.45)	1.69 (1.59–1.80)
5–8 relatives	3.97 (3.75–4.20)	2.10 (1.96–2.24)
9 or more relatives	5.61 (5.27–5.97)	2.34 (2.17–2.51)
**Number of friends**		
0 friends	Ref.	Ref.
1 friend	1.35 (1.28–1.43)	1.34 (1.26–1.42)
2 friends	1.99 (1.89–2.10)	1.79 (1.69–1.89)
3 or 4 friends	3.36 (3.20–3.53)	2.65 (2.52–2.79)
5–8 friends	5.69 (5.41–5.98)	3.88 (3.67–4.10)
9 or more friends	8.51 (8.08–8.96)	5.22 (4.92–5.53)
**Having pet**		
No	Ref.	Ref.
Yes	1.03 (1.00–1.05)	1.24 (1.21–1.27)
**Number of household members**		
0 members	Ref.	Ref.
1 member	1.59 (1.54–1.63)	1.05 (1.00–1.09)
2–3 members	1.11 (1.07–1.14)	1.13 (1.08–1.19)
4–5 members	0.92 (0.88–0.97)	1.09 (1.02–1.17)
> 5 members	0.60 (0.54–0.67)	0.98 (0.86–1.11)
**Children**		
No	Ref.	Ref.
Yes	1.85 (1.80–1.89)	0.93 (0.90–0.96)
**Disability**		
No	Ref.	Ref.
Yes	0.50 (0.48–0.51)	0.77 (0.74–0.80)
Would rather not say	0.39 (0.36–0.42)	0.68 (0.62–0.74)
**Longterm conditions**		
No	Ref.	Ref.
Yes	0.56 (0.55–0.58)	0.82 (0.80–0.85)
Would rather not say	0.45 (0.41–0.48)	0.75 (0.69–0.82)

Note: *Adjusted models included age*,* gender*,* ethnicity*,* education*,* employment*,* marital status*,* social contacts*,* household size*,* pet ownership*,* children*,* disability and long-term conditions. Model-based intervals are conditional on the fitted model and analysed respondents; they do not account for uncertainty or bias arising from non-probability selection and do not support population-level inference. Abbreviations: OR*,* odds ratio; aOR*,* adjusted odds ratio; MBI*,* model-based interval; N*,* number of observations; Ref.*,* reference category; no further abbreviations*

Social contact remained strongly associated with social capital. Relative to respondents reporting no friends, those reporting progressively more friends had increasingly higher adjusted odds of high social capital; respondents reporting nine or more friends had more than fivefold higher adjusted odds (aOR = 5.22, 95% MBI: 4.92–5.53). A similar graded pattern was observed relative to respondents reporting no relatives.

Health-related factors were inversely associated with social capital. Relative to respondents reporting no disability, respondents with a disability had lower adjusted odds of high social capital (aOR = 0.77, 95% MBI: 0.74–0.80). Relative to respondents reporting no long-term condition, those with a long-term condition also had lower adjusted odds (aOR = 0.82, 95% MBI: 0.80–0.85).

Social capital was dichotomised at the median. Models adjust for age, gender, ethnicity, education, employment, marital status, social contacts, household size, pet ownership, children, disability and long-term conditions. Reference categories are shown. Values > 1 indicate higher odds of high social capital.

### Converging factors associated with loneliness and social capital

Across the loneliness models, younger age, unemployment, disability and limited social contact were consistently associated with higher loneliness. In the social-capital model, younger age, unemployment and disability were associated with lower odds of high social capital, whereas more frequent contact with friends and relatives was associated with higher odds of high social capital. Marital-status patterns differed across outcomes: married or civil-partnered respondents had lower loneliness and higher odds of high social capital, whereas widowed respondents had higher loneliness but also higher odds of high social capital relative to single respondents.

Social capital was itself socially patterned, being more frequently reported among older, married and retired respondents. Some divergences were also observed: university education was associated with higher loneliness in the UCLA-3 model but lower loneliness in the DMOL model and higher odds of high social capital. These differences may reflect the distinct constructs and measurement approaches captured by the three outcomes, as well as unmeasured contextual factors such as occupational stress or urbanicity.

## Discussion

Drawing on data from over 135,000 adults, this study presents a large analysis of adjusted associations with loneliness and social capital within a non-probability sample in the UK. Building on the descriptive characterisations presented in our previous work [6] this second paper estimates adjusted associations and generates hypotheses for future targeted, equity-informed and context-sensitive intervention research. However, given the non-probability sampling design, regression estimates represent within-sample associations rather than population-level effects and should be interpreted as conditional relationships within the responding cohort and as hypothesis-generating rather than generalisable estimates.

Using two validated tools (UCLA-3 and DMOL), we identified clear sociodemographic and social gradients in loneliness. Younger adults, particularly those aged 16–25, reported the highest observed levels of loneliness, while older adults generally reported lower levels. However, within the older respondent group (≥ 65), important disparities were observed, especially among those who were widowed, living alone or managing chronic health conditions. Social contact, measured by the number of close friends or relatives, a known indicator of social connection, emerged as one of the strongest and most consistent factors associated with lower loneliness across both models. Social capital, conceptualised as neighbourhood trust, cohesion and reciprocity, was examined as a separate contextual outcome and was more frequently reported among older, married and retired respondents. Its inclusion alongside the loneliness outcomes draws from the social determinants of health framework advocated by the World Health Organization (WHO) [17, 18]. These findings describe how perceived neighbourhood trust, cohesion and reciprocity varied across respondent characteristics but do not establish causal effects on individuals’ social relationships.

Among older respondents, retirement and stable relationships were associated with lower loneliness in the adjusted models; however, these estimates should not be interpreted as protective effects, and age-stratified analyses are needed to assess whether these associations vary across age groups. However, this observed pattern was not uniformly distributed since older individuals who were widowed, living alone or experiencing multimorbidity more frequently reported higher loneliness, echoing findings from the English Longitudinal Study of Ageing (ELSA) [19]. This highlights the need to disaggregate the “older adult” category and address intra-cohort heterogeneity in loneliness trajectories.

Collectively, these findings emphasise age-related differences in loneliness within a cross-sectional sample and support age-sensitive approaches, while longitudinal data are needed to examine trajectories over time. Nevertheless, our findings align with socioecological models of health which emphasise that loneliness is shaped by multilevel influences, including individual traits, interpersonal ties, community environments and structural forces [20, 21]. While individual factors such as age, disability or employment status matter, they operate within broader systems of opportunity, access and belonging. Consistent with with our previous work[ 6] which demonstrated substantial heterogeneity in loneliness across subgroups, the present analysis quantifies these gradients and identifies converging associations across multiple measures. However, we emphasise that these findings reflect cross-sectional associations and do not establish causal or mediating relationships.

Our results are consistent with prior evidence showing a reversed age-loneliness gradient in the general population, where younger adults report greater loneliness than older adults [22, 23]. Our study’s large sample size brings new insight into the dual burden of loneliness in later life, where some older individuals demonstrate resilience, while others, particularly those who are widowed, isolated or unwell, may experience comparatively high loneliness.

The strong inverse association between social contact and loneliness aligns with longitudinal findings from ELSA and international reviews [24]. Within this sample, the models quantify this gradient and show that individuals reporting a greater number of social ties (e.g. more than four friends or relatives) had substantially lower odds of loneliness.

In relation to older adults, our findings echo those of Victor et al. in 2005 [25], who distinguished between emotional loneliness (absence of close attachment) and social loneliness (lack of broader networks). Although this distinction was not captured directly by our measures, it was evident in subgroup patterns and could help explain why older adults who are widowed but socially embedded may experience less social loneliness but elevated emotional loneliness.

Intriguingly, the observed association between higher educational attainment and increased loneliness on the UCLA measure contrasts with prior studies linking higher education to improved social and mental health outcomes[26]. This pattern may also be interpreted through expectation-discrepancy frameworks whereby individuals with greater educational attainment may hold higher expectations of social connectedness, increasing the likelihood of perceived deficits despite comparable objective networks. The observed divergence may also reflect a combination of measurement differences between indirect (UCLA) and direct (DMOL) instruments, as well as underlying contextual and structural factors. For example, higher educational attainment may be associated with different expectations of social connectedness, patterns of social mobility or the composition and stability of social networks. It may also reflect contextual influences such as work-related demands, urban residence, digitally mediated forms of interaction or differences in neighbourhood integration among more highly educated individuals [27]. However, these mechanisms cannot be directly assessed within the present analysis and should be interpreted cautiously.

The slightly elevated loneliness observed among individuals with children-particularly in the DMOL model, may reflect caregiving-related circumstances; however, these mechanisms were not measured. One hypothesis is that caregiving responsibilities may reduce time for reciprocal adult relationships, particularly alongside single parenting, chronic illness or financial stress. The absence of an adjusted association between pet ownership and loneliness should not be interpreted as evidence that companion animals do not support social connection, because any association may be context-specific [28]. We hypothesise that these dynamics may be more salient among older adults who live alone, highlighting the need for future research to differentiate between emotional and structural sources of connection.

This study’s primary strength lies in its scale and scope, representing the largest UK-based analysis of loneliness and social capital to date. It includes validated measures of loneliness and adopts a novel approach by examining social capital as a complementary contextual outcome alongside two loneliness outcomes, thus enabling a more integrated assessment of relational and structural dimensions of social connection. The analysis also provides age-disaggregated insights, highlighting both resilience and vulnerability among older adults. However, several limitations should be considered, some of which are detailed in our previous work [6].

The principal limitations of this study are the non-probability sampling design, which limits generalisability and does not support population-level inference, and the cross-sectional design, which precludes causal inference. Because participation was voluntary and individual inclusion probabilities were unknown, the direction and magnitude of selection bias cannot be quantified. The large sample size reduces random error but does not correct systematic selection bias; narrow model-based intervals therefore do not establish population representativeness. Associations may differ from those in the wider English population if participation was related to both the covariates and loneliness outcomes. Covariate adjustment and multiple imputation cannot restore representativeness or remove bias from unmeasured selection mechanisms. Temporal ordering cannot be established, and reverse causation and residual confounding remain plausible; the aORs should therefore not be interpreted as causal effects or measures of predictive performance. Self-reported data may introduce recall and social desirability bias, and online sampling may underrepresent individuals with cognitive or digital access barriers, particularly among older adults. Additionally, variables such as income and more granular measures of socioeconomic position were not included and may interact with loneliness, warranting further investigation.

Importantly, self-selection may affect not only the observed distribution of loneliness but also the estimated associations between loneliness and the included covariates. If participation was jointly related to loneliness and respondent characteristics, the magnitude (and potentially the direction) of the adjusted associations may differ from those in the wider population. The regression models should therefore be understood as parsimonious summaries of conditional patterns among INTERACT respondents rather than estimates of relationships that necessarily transport to adults in England.

A further limitation is the reliance on established, yet inherently constrained measures of loneliness. While the UCLA-3 and ONS single-item scales were selected to ensure comparability with existing national datasets, they do not fully capture the multidimensional nature of loneliness. The UCLA scale, originally developed within a specific demographic context, provides limited insight into duration, contextual drivers and experiential aspects of loneliness. Similarly, the ONS measure captures frequency but does not specify a timeframe or underlying causes. Further, collapsing the UCLA-3 measure into broader ordered categories improved comparability with the DMOL measure but reduced the variability available for analysis and may have obscured more subtle gradients in loneliness severity. Future research should prioritise the development and validation of contemporary, multidimensional measures that better reflect the complexity and lived experience of loneliness across diverse populations. These limitations are addressed in part through ongoing psychometric work reported elsewhere [12], and through parallel efforts to develop and validate novel measures of social connection (the INTERACTION Scale) as will be reported in a forthcoming paper elsewhere.

We acknowledge that the absence of area-level contextual variables, such as rural-urban classification and neighbourhood deprivation within the present analytical models is also a limitation. These factors are recognised as important structural determinants of loneliness and may contribute to geographic and socioeconomic inequalities in social connectedness. While such variables were available within the INTERACT dataset and incorporated into geospatial analyses, they were not included in the current modelling framework. This reflects the intentional focus of this paper on individual-level socio-demographic, social and health-related correlates to provide a clear and interpretable analytical baseline. More detailed examination of area-level determinants, including rurality and deprivation, is being undertaken in complementary analyses within the INTERACT programme.

Another important limitation is the reliance on main-effects models which estimate mutually adjusted associations but do not capture the combined influence of intersecting socio-demographic characteristics. Although interaction modelling was considered, the inclusion of multiple higher-order terms across numerous covariates in a large dataset risks over-parameterisation, multicollinearity and reduced interpretability. Accordingly, the present analysis does not account for the compounded or intersectional nature of vulnerability, where co-occurring characteristics (e.g. age, disability and socioeconomic status) may interact in non-additive ways. Future research should apply theoretically specified interaction models or alternative approaches such as latent class or intersectional frameworks to better capture these complex patterns. Furthermore, the use of regression models within a non-probability sample does not support population-level inference and observed associations may reflect underlying selection mechanisms rather than true population relationships.

Finally, although this analysis focuses on individual-level determinants to provide interpretable baseline estimates, area-level factors, including rural-urban classification and neighbourhood deprivation, represent important contextual extensions. These are examined in complementary analyses within the wider INTERACT programme (manuscript in preparation).

Given the cross-sectional, non-probability sampling design, these findings do not provide a direct basis for policy prescription, resource allocation or population-level prioritisation. The recalculated descriptive proportions and adjusted models identify patterns within the responding sample rather than population prevalence estimates or causal effects. Accordingly, the observed associations should be treated as hypotheses about groups and contextual factors that may warrant further investigation.

The consistency of several within-sample associations across the UCLA-3 and DMOL measures, particularly those involving age, marital status, social contact, employment and health, provides a rationale for their examination in representative and longitudinal research. Future studies should establish whether these patterns persist in probability-based or appropriately calibrated samples, assess temporal ordering and investigate the extent to which observed differences reflect selection, measurement, confounding or potentially modifiable mechanisms.

Intervention and policy evaluations could subsequently test whether approaches intended to strengthen social connection, neighbourhood cohesion or access to community infrastructure improve loneliness-related outcomes, for whom and under what circumstances. Such approaches may include inclusive public infrastructure, intergenerational programmes, community hubs, group-based activities, neighbourhood walking groups and digital-literacy initiatives, which have previously shown promise in reducing social isolation and improving wellbeing in later life [29]. Their inclusion here should be understood as identifying candidates for prospective evaluation, rather than as recommendations arising directly from the present study.

Future evaluations could also examine whether place-based approaches aligned with the WHO Age-Friendly Communities framework improve social connection across different population groups. These evaluations should use prospective designs, clearly specified comparison groups and equity-sensitive analyses, and should assess potential differential effects across age, socioeconomic and health-related groups.

Further research should also strengthen the measurement of loneliness and social capital across diverse populations. The subsequent INTERACT psychometric analysis applies Rasch modelling to examine item functioning, scale reliability, response-category performance and differential item functioning across key sociodemographic groups [12].

This work provides an important complementary basis for determining whether observed subgroup differences reflect substantive variation, measurement properties or both. Until representative, longitudinal and intervention-based evidence is available, the present findings are best understood as informing research priorities, measurement development and intervention hypotheses rather than supporting specific policy actions.

## Conclusions

Loneliness is a complex and layered experience that reflects individual disposition, as well as structural and relational disadvantage. This study provides large-sample estimates of adjusted within-sample associations with loneliness among study respondents. Findings highlight consistent gradients across age, social relationships and health status, and underscore the importance of considering both individual and contextual dimensions of social disconnection. These hypothesis-generating results provide a foundation for future longitudinal and intervention-focused research. These findings should not be interpreted as population estimates, causal effects or confirmatory evidence.

## Supplementary information

Below is the link to the electronic supplementary material.

Supplementary Material 1 : Survey instrument. Original INTERACT questionnaire, including consent, demographic and study items.

Supplementary Material 2 : Measure wording and scoring. Tables S1–S3: loneliness and social-capital item wording, response options and scoring.

Supplementary Material 3 : Descriptive distributions. Tables S4–S6: within-sample UCLA-3, DMOL and social-capital distributions, combined into one file.

Supplementary Material 4 : Multiple-imputation sensitivity analysis. Figs S1–S5 and Tables S7–S9: missingness, convergence diagnostics and pooled regression estimates.

Supplementary Material 5 : STROBE checklist. Table S10: cross-sectional reporting checklist with manuscript locations and reporting limitations.

Supplementary Material 6 : CHERRIES checklist. Table S11: internet-survey reporting checklist with source locations and unavailable procedural information.

## Data Availability

Due to ethical and data protection constraints, individual-level data cannot be publicly shared. A de-identified dataset and accompanying codebook can be made available upon reasonable request from the corresponding author, subject to institutional approvals and data protection requirements.

## Declarations

### Twitter

@austenelosta @ImperialSCARU @LonelinessStudy.
