# Supplementary material for: Factors associated with loneliness and social capital in a large volunteer sample of adults in England
Source: BMC Glob Public Health. 2026 Oct 2;4:96. doi: 10.1186/s44263-026-00335-1 (PMC13632327; doi:10.1186/s44263-026-00335-1)
Supplement: Supplementary file 2 [file 44263_2026_335_MOESM2_ESM.docx]

Supplementary material 2: Measure wording, response options and scoring

This supplementary file provides the wording of the loneliness and social-capital items used in the INTERACT study, together with their response options and the scoring procedures applied in the present analysis.

# Three-Item Loneliness Scale

**Stem:** How often do you feel...

Table S1: UCLA-3 item wording and scoring

| **Item** | **Complete item wording** | **Response options** | **Item scoring** |
| --- | --- | --- | --- |
| 1 | How often do you feel that you lack companionship? | Hardly ever or never Some of the time Often | 1 2 3 |
| 2 | How often do you feel left out? | Hardly ever or never Some of the time Often | 1 2 3 |
| 3 | How often do you feel isolated from others? | Hardly ever or never Some of the time Often | 1 2 3 |

Abbreviations: UCLA, University of California, Los Angeles; DMOL, Direct Measure of Loneliness; ONS, Office for National Statistics; N/n, number of respondents.

**Scoring procedure.** The three item scores were summed to produce a total score ranging from 3 to 9, with higher scores indicating more frequent loneliness. For the present analyses, totals were grouped into three ordered categories: **low/no loneliness = 3; moderate loneliness = 4–6; high/severe loneliness = 7–9.**

# ONS Direct Measure of Loneliness (DMOL)

Table S2: DMOL item wording and analytic categories

| **Item** | **Complete item wording** | **Response options** | **Analytic category** |
| --- | --- | --- | --- |
| 1 | How often do you feel lonely? | Never | Low |
|  |  | Hardly ever | Low |
|  |  | Occasionally | Moderate |
|  |  | Some of the time | Moderate |
|  |  | Often or always | High/severe |

Abbreviations: UCLA, University of California, Los Angeles; DMOL, Direct Measure of Loneliness; ONS, Office for National Statistics; N/n, number of respondents.

**Scoring procedure.** The five ordered response categories were collapsed into three ordered categories for analysis: **low = never/hardly ever; moderate = occasionally/some of the time; high/severe = often or always.** The ordered outcome was modelled using ordinal logistic regression, with higher categories representing more frequent loneliness.

**Seven-item social-capital scale**

**Instruction:** To what extent do you agree or disagree with the following statements?

Table S3: Social-capital item wording and scoring

| **Item** | **Complete item wording** | **Polarity** | **Response options** | **Binary scoring** |
| --- | --- | --- | --- | --- |
| 1 | People around here are willing to help their neighbours. | Positive | Strongly disagree Disagree Agree Strongly agree | 0 0 1 1 |
| 2 | This is a close-knit, or “tight”, neighbourhood where people generally know one another. | Positive | Strongly disagree Disagree Agree Strongly agree | 0 0 1 1 |
| 3 | If I had to borrow £30 in an emergency, I could borrow it from a neighbour. | Positive | Strongly disagree Disagree Agree Strongly agree | 0 0 1 1 |
| 4 | People in this neighbourhood generally don’t get along with each other. | Negative | Strongly disagree Disagree Agree Strongly agree | 1 1 0 0 |
| 5 | People in this neighbourhood can be trusted. | Positive | Strongly disagree Disagree Agree Strongly agree | 0 0 1 1 |
| 6 | If I were sick, I could count on my neighbours to shop for groceries for me. | Positive | Strongly disagree Disagree Agree Strongly agree | 0 0 1 1 |
| 7 | People in this neighbourhood do not share the same values. | Negative | Strongly disagree Disagree Agree Strongly agree | 1 1 0 0 |

Abbreviations: UCLA, University of California, Los Angeles; DMOL, Direct Measure of Loneliness; ONS, Office for National Statistics; N/n, number of respondents.

**Scoring procedure.** For positively worded items, “agree” and “strongly agree” were scored 1, while “disagree” and “strongly disagree” were scored 0. The two negatively worded items (Items 4 and 7) were reverse-coded, so that disagreement was scored 1 and agreement was scored 0. Item scores were summed to generate an index ranging from 0 to 7, with higher values indicating greater perceived neighbourhood social capital. For the regression analysis, the summed score was **dichotomised as low social capital = 0–4 and high social capital = 5–7 (>4).**

# General analytic notes

**•** All measure scores were calculated using a classical test theory approach based on item summation.

**•** Outcome-specific analyses used available complete cases; respondents with missing data required for a given score were excluded from that outcome’s complete-case analysis.

**•** The UCLA/Three-Item Loneliness Scale and DMOL outcomes were analysed as ordered outcomes. The dichotomised social-capital score was analysed using binary logistic regression.

**•** The loneliness severity groupings and the social-capital cut-point were pragmatic analytic classifications used in this study and should not be interpreted as diagnostic or universally established thresholds.

# Reliability assessment

Internal consistency was assessed using Cronbach's α and McDonald's ω. For the binary-scored social-capital items used in the regression analyses, reliability was additionally quantified using Kuder–Richardson Formula 20 (KR-20). Ordinal reliability estimates based on polychoric correlations were also calculated.

Abbreviations: UCLA, University of California, Los Angeles; ONS, Office for National Statistics; DMOL, Direct Measure of Loneliness; KR-20, Kuder–Richardson Formula 20.
