# Supplementary material for: Factors associated with loneliness and social capital in a large volunteer sample of adults in England
Source: BMC Glob Public Health. 2026 Oct 2;4:96. doi: 10.1186/s44263-026-00335-1 (PMC13632327; doi:10.1186/s44263-026-00335-1)
Supplement: Supplementary file 3 [file 44263_2026_335_MOESM3_ESM.docx]

Supplementary material 3: Descriptive distributions

Table S4: Distribution of UCLA-3 loneliness categories within respondent characteristics among INTERACT participants

| **Respondent characteristic** | **Respondents**  **n** | **UCLA-3 loneliness category** | | |
| --- | --- | --- | --- | --- |
|  |  | **Low/no loneliness**  **n (%)** | **Moderate loneliness**  **n (%)** | **Severe loneliness**  **n (%)** |
| **Overall sample** | **135,722** | **38,728 (28.5)** | **55,626 (41.0)** | **41,368 (30.5)** |
| **Age** | | | | |
| 16-25 | 8,352 | 583 (7.0) | 3,183 (38.1) | 4,586 (54.9) |
| 26-35 | 13,057 | 1,502 (11.5) | 5,534 (42.4) | 6,021 (46.1) |
| 36-45 | 15,006 | 2,392 (15.9) | 6,178 (41.2) | 6,436 (42.9) |
| 46-55 | 22,031 | 4,852 (22.0) | 9,062 (41.1) | 8,117 (36.8) |
| 56-65 | 33,235 | 10,525 (31.7) | 13,720 (41.3) | 8,990 (27.0) |
| >65 | 43,927 | 18,845 (42.9) | 17,899 (40.7) | 7,183 (16.4) |
| *Missing* | *114* | *29 (25.4)* | *50 (43.9)* | *35 (30.7)* |
| **Gender** | | | | |
| Female | 83,661 | 21,588 (25.8) | 36,102 (43.2) | 25,971 (31.0) |
| Male | 50,729 | 16,983 (33.5) | 19,092 (37.6) | 14,654 (28.9) |
| Other | 804 | 69 (8.6) | 246 (30.6) | 489 (60.8) |
| Would rather not say | 498 | 82 (16.5) | 173 (34.7) | 243 (48.8) |
| *Missing* | *30* | *6 (20.0)* | *13 (43.3)* | *11 (36.7)* |
| **Education** | | | | |
| A levels/College | 36,700 | 9,896 (27.0) | 14,645 (39.9) | 12,159 (33.1) |
| Secondary School | 30,481 | 8,758 (28.7) | 11,419 (37.5) | 10,304 (33.8) |
| University Degree or higher | 58,008 | 17,632 (30.4) | 25,288 (43.6) | 15,088 (26.0) |
| *Missing* | *10,533* | *2,442 (23.2)* | *4,274 (40.6)* | *3,817 (36.2)* |
| **Employment** | | | | |
| Employed full-time | 38,388 | 8,777 (22.9) | 17,152 (44.7) | 12,459 (32.5) |
| Employed part-time | 14,604 | 3,928 (26.9) | 6,663 (45.6) | 4,013 (27.5) |
| Furloughed | 54 | 3 (5.6) | 14 (25.9) | 37 (68.5) |
| Other | 4,083 | 410 (10.0) | 1,114 (27.3) | 2,559 (62.7) |
| Retired | 45,038 | 18,961 (42.1) | 18,303 (40.6) | 7,774 (17.3) |
| Self-employed | 7,608 | 2,443 (32.1) | 3,140 (41.3) | 2,025 (26.6) |
| Student (full or part-time) | 3,733 | 305 (8.2) | 1,533 (41.1) | 1,895 (50.8) |
| Unemployed | 7,629 | 461 (6.0) | 1,855 (24.3) | 5,313 (69.6) |
| Unpaid carer | 1,560 | 150 (9.6) | 537 (34.4) | 873 (56.0) |
| Volunteer (full or part-time) | 1,459 | 507 (34.7) | 601 (41.2) | 351 (24.1) |
| *Missing* | *11,566* | *2,783 (24.1)* | *4,714 (40.8)* | *4,069 (35.2)* |
| **Ethnicity** | | | | |
| Asian/Asian British | 5,162 | 907 (17.6) | 2,161 (41.9) | 2,094 (40.6) |
| British Black/African/Caribbean | 2,310 | 434 (18.8) | 941 (40.7) | 935 (40.5) |
| Mixed/Multiple ethnic groups | 2,025 | 387 (19.1) | 770 (38.0) | 868 (42.9) |
| Other ethnic group | 2,722 | 603 (22.2) | 1,030 (37.8) | 1,089 (40.0) |
| White | 112,164 | 33,752 (30.1) | 46,104 (41.1) | 32,308 (28.8) |
| White and Black Caribbean | 462 | 72 (15.6) | 166 (35.9) | 224 (48.5) |
| *Missing* | *10,877* | *2,573 (23.7)* | *4,454 (40.9)* | *3,850 (35.4)* |
| **Marital Status** | | | | |
| Divorced | 12,761 | 2,280 (17.9) | 5,256 (41.2) | 5,225 (40.9) |
| In a relationship | 12,404 | 3,017 (24.3) | 5,497 (44.3) | 3,890 (31.4) |
| Married / Civil partnership | 60,612 | 25,470 (42.0) | 25,150 (41.5) | 9,992 (16.5) |
| Other | 2,992 | 466 (15.6) | 1,146 (38.3) | 1,380 (46.1) |
| Widowed | 9,625 | 2,022 (21.0) | 4,546 (47.2) | 3,057 (31.8) |
| Single | 26,983 | 3,059 (11.3) | 9,793 (36.3) | 14,131 (52.4) |
| *Missing* | *10,345* | *2,414 (23.3)* | *4,238 (41.0)* | *3,693 (35.7)* |
| **Having relatives** | | | | |
| 0 | 7,195 | 959 (13.3) | 2,255 (31.3) | 3,981 (55.3) |
| 1 | 12,101 | 1,586 (13.1) | 4,120 (34.0) | 6,395 (52.8) |
| 2 | 20,994 | 2,949 (14.0) | 8,500 (40.5) | 9,545 (45.5) |
| 3 or 4 | 39,644 | 9,703 (24.5) | 17,947 (45.3) | 11,994 (30.3) |
| 5-8 | 29,656 | 12,201 (41.1) | 12,961 (43.7) | 4,494 (15.2) |
| 9 or more | 15,825 | 8,923 (56.4) | 5,619 (35.5) | 1,283 (8.1) |
| *Missing* | *10,307* | *2,407 (23.4)* | *4,224 (41.0)* | *3,676 (35.7)* |
| **Having friends** | | | | |
| 0 | 11,936 | 1,022 (8.6) | 3,363 (28.2) | 7,551 (63.3) |
| 1 | 13,850 | 1,193 (8.6) | 4,735 (34.2) | 7,922 (57.2) |
| 2 | 19,188 | 2,613 (13.6) | 8,078 (42.1) | 8,497 (44.3) |
| 3 or 4 | 31,679 | 7,917 (25.0) | 15,035 (47.5) | 8,727 (27.5) |
| 5-8 | 25,299 | 10,207 (40.3) | 11,663 (46.1) | 3,429 (13.6) |
| 9 or more | 23,465 | 13,370 (57.0) | 8,524 (36.3) | 1,571 (6.7) |
| *Missing* | *10,305* | *2,406 (23.3)* | *4,228 (41.0)* | *3,671 (35.6)* |
| **Having pets** | | | | |
| No | 69,895 | 21,728 (31.1) | 28,491 (40.8) | 19,676 (28.2) |
| Yes | 55,413 | 14,566 (26.3) | 22,875 (41.3) | 17,972 (32.4) |
| *Missing* | *10,414* | *2,434 (23.4)* | *4,260 (40.9)* | *3,720 (35.7)* |
| **Household size** | | | | |
| 0 members | 33,489 | 5,948 (17.8) | 13,673 (40.8) | 13,868 (41.4) |
| 1 member | 52,760 | 20,257 (38.4) | 21,236 (40.3) | 11,267 (21.4) |
| 2-3 members | 30,407 | 8,075 (26.6) | 12,936 (42.5) | 9,396 (30.9) |
| 4-5 members | 7,278 | 1,773 (24.4) | 3,008 (41.3) | 2,497 (34.3) |
| > 5 members | 1,494 | 280 (18.7) | 546 (36.5) | 668 (44.7) |
| *Missing* | *10,294* | *2,395 (23.3)* | *4,227 (41.1)* | *3,672 (35.7)* |
| **Having children** | | | | |
| No | 40,684 | 7,563 (18.6) | 16,783 (41.3) | 16,338 (40.2) |
| Yes | 84,561 | 28,717 (34.0) | 34,557 (40.9) | 21,287 (25.2) |
| *Missing* | *10,477* | *2,448 (23.4)* | *4,286 (40.9)* | *3,743 (35.7)* |
| **Having children under 16** | | | | |
| No | 64,991 | 24,467 (37.6) | 26,305 (40.5) | 14,219 (21.9) |
| Yes | 19,287 | 4,160 (21.6) | 8,121 (42.1) | 7,006 (36.3) |
| *Missing* | *283* | *90 (31.8)* | *131 (46.3)* | *62 (21.9)* |
| **Having disability** | | | | |
| No | 96,817 | 32,497 (33.6) | 41,613 (43.0) | 22,707 (23.5) |
| Prefer not to say | 3,584 | 436 (12.2) | 1,295 (36.1) | 1,853 (51.7) |
| Yes | 24,915 | 3,364 (13.5) | 8,450 (33.9) | 13,101 (52.6) |
| *Missing* | *10,406* | *2,431 (23.4)* | *4,268 (41.0)* | *3,707 (35.6)* |
| **Having long-term conditions** | | | | |
| No | 60,260 | 23,149 (38.4) | 26,162 (43.4) | 10,949 (18.2) |
| Prefer not to say | 3,085 | 558 (18.1) | 1,204 (39.0) | 1,323 (42.9) |
| Yes | 62,038 | 12,604 (20.3) | 24,023 (38.7) | 25,411 (41.0) |
| *Missing* | *10,339* | *2,417 (23.4)* | *4,237 (41.0)* | *3,685 (35.6)* |

Abbreviations: UCLA, University of California, Los Angeles; DMOL, Direct Measure of Loneliness; ONS, Office for National Statistics; N/n, number of respondents.

**Notes:** Percentages are row percentages and therefore sum to approximately 100% across the three loneliness categories for each respondent subcategory. Findings describe the distribution observed among INTERACT participants and should not be interpreted as population prevalence estimates. The total analytical sample for this table was N=135,722. Missing denotes respondents with no recorded value for the relevant characteristic.

Table S5: Distribution of DMOL loneliness categories within respondent characteristics among INTERACT participants

| **Respondent characteristic** | **Respondents n** | **DMOL category** | | |
| --- | --- | --- | --- | --- |
|  |  | **No loneliness n (%)** | **Moderate loneliness n (%)** | **Severe loneliness n (%)** |
| **Overall sample** | **135,722** | **46,355 (34.2)** | **67,001 (49.4)** | **22,366 (16.5)** |
| **Age** | | | | |
| 16-25 | 8,352 | 1,015 (12.2) | 4,821 (57.7) | 2,516 (30.1) |
| 26-35 | 13,057 | 2,289 (17.5) | 7,447 (57.0) | 3,321 (25.4) |
| 36-45 | 15,006 | 3,387 (22.6) | 8,023 (53.5) | 3,596 (24.0) |
| 46-55 | 22,031 | 6,210 (28.2) | 11,390 (51.7) | 4,431 (20.1) |
| 56-65 | 33,235 | 12,532 (37.7) | 16,098 (48.4) | 4,605 (13.9) |
| >65 | 43,927 | 20,886 (47.5) | 19,172 (43.6) | 3,869 (8.8) |
| *Missing* | *114* | *36 (31.6)* | *50 (43.9)* | *28 (24.6)* |
| **Gender** | | | | |
| Female | 83,661 | 25,536 (30.5) | 44,378 (53.0) | 13,747 (16.4) |
| Male | 50,729 | 20,565 (40.5) | 21,978 (43.3) | 8,186 (16.1) |
| Other | 804 | 137 (17.0) | 384 (47.8) | 283 (35.2) |
| Would rather not say | 498 | 111 (22.3) | 246 (49.4) | 141 (28.3) |
| *Missing* | *30* | *6 (20.0)* | *15 (50.0)* | *9 (30.0)* |
| **Education** | | | | |
| A levels/College | 36,700 | 11,871 (32.3) | 18,316 (49.9) | 6,513 (17.7) |
| Secondary School | 30,481 | 9,746 (32.0) | 14,629 (48.0) | 6,106 (20.0) |
| University Degree or higher | 58,008 | 22,033 (38.0) | 28,548 (49.2) | 7,427 (12.8) |
| *Missing* | *10,533* | *2,705 (25.7)* | *5,508 (52.3)* | *2,320 (22.0)* |
| **Employment** | | | | |
| Employed full-time | 38,388 | 11,558 (30.1) | 20,618 (53.7) | 6,212 (16.2) |
| Employed part-time | 14,604 | 4,796 (32.8) | 7,774 (53.2) | 2,034 (13.9) |
| Furloughed | 54 | 8 (14.8) | 25 (46.3) | 21 (38.9) |
| Other | 4,083 | 586 (14.4) | 1,953 (47.8) | 1,544 (37.8) |
| Retired | 45,038 | 21,339 (47.4) | 19,582 (43.5) | 4,117 (9.1) |
| Self-employed | 7,608 | 2,928 (38.5) | 3,670 (48.2) | 1,010 (13.3) |
| Student (full or part-time) | 3,733 | 536 (14.4) | 2,250 (60.3) | 947 (25.4) |
| Unemployed | 7,629 | 687 (9.0) | 3,546 (46.5) | 3,396 (44.5) |
| Unpaid carer | 1,560 | 223 (14.3) | 900 (57.7) | 437 (28.0) |
| Volunteer (full or part-time) | 1,459 | 610 (41.8) | 667 (45.7) | 182 (12.5) |
| *Missing* | *11,566* | *3,084 (26.7)* | *6,016 (52.0)* | *2,466 (21.3)* |
| **Ethnicity** | | | | |
| Asian/Asian British | 5,162 | 1,148 (22.2) | 2,764 (53.5) | 1,250 (24.2) |
| British Black/African/Caribbean | 2,310 | 511 (22.1) | 1,263 (54.7) | 536 (23.2) |
| Mixed/Multiple ethnic groups | 2,025 | 488 (24.1) | 1,078 (53.2) | 459 (22.7) |
| Other ethnic group | 2,722 | 721 (26.5) | 1,311 (48.2) | 690 (25.3) |
| White | 112,164 | 40,525 (36.1) | 54,653 (48.7) | 16,986 (15.1) |
| White and Black Caribbean | 462 | 99 (21.4) | 226 (48.9) | 137 (29.7) |
| *Missing* | *10,877* | *2,863 (26.3)* | *5,706 (52.5)* | *2,308 (21.2)* |
| **Marital Status** | | | | |
| Divorced | 12,761 | 2,915 (22.8) | 7,030 (55.1) | 2,816 (22.1) |
| In a relationship | 12,404 | 4,017 (32.4) | 6,682 (53.9) | 1,705 (13.7) |
| Married / Civil partnership | 60,612 | 29,623 (48.9) | 26,007 (42.9) | 4,982 (8.2) |
| Other | 2,992 | 594 (19.9) | 1,592 (53.2) | 806 (26.9) |
| Widowed | 9,625 | 2,168 (22.5) | 5,764 (59.9) | 1,693 (17.6) |
| Single | 26,983 | 4,362 (16.2) | 14,488 (53.7) | 8,133 (30.1) |
| *Missing* | *10,345* | *2,676 (25.9)* | *5,438 (52.6)* | *2,231 (21.6)* |
| **Having relatives** | | | | |
| 0 | 7,195 | 1,362 (18.9) | 3,359 (46.7) | 2,474 (34.4) |
| 1 | 12,101 | 2,188 (18.1) | 6,316 (52.2) | 3,597 (29.7) |
| 2 | 20,994 | 4,074 (19.4) | 11,958 (57.0) | 4,962 (23.6) |
| 3 or 4 | 39,644 | 11,944 (30.1) | 21,794 (55.0) | 5,906 (14.9) |
| 5-8 | 29,656 | 14,219 (47.9) | 13,091 (44.1) | 2,346 (7.9) |
| 9 or more | 15,825 | 9,897 (62.5) | 5,057 (32.0) | 871 (5.5) |
| *Missing* | *10,307* | *2,671 (25.9)* | *5,426 (52.6)* | *2,210 (21.4)* |
| **Having friends** | | | | |
| 0 | 11,936 | 1,667 (14.0) | 5,826 (48.8) | 4,443 (37.2) |
| 1 | 13,850 | 1,872 (13.5) | 7,712 (55.7) | 4,266 (30.8) |
| 2 | 19,188 | 3,717 (19.4) | 11,295 (58.9) | 4,176 (21.8) |
| 3 or 4 | 31,679 | 9,964 (31.5) | 17,533 (55.3) | 4,182 (13.2) |
| 5-8 | 25,299 | 11,779 (46.6) | 11,624 (45.9) | 1,896 (7.5) |
| 9 or more | 23,465 | 14,684 (62.6) | 7,584 (32.3) | 1,197 (5.1) |
| *Missing* | *10,305* | *2,672 (25.9)* | *5,427 (52.7)* | *2,206 (21.4)* |
| **Having pets** | | | | |
| No | 69,895 | 25,580 (36.6) | 33,424 (47.8) | 10,891 (15.6) |
| Yes | 55,413 | 18,079 (32.6) | 28,109 (50.7) | 9,225 (16.6) |
| *Missing* | *10,414* | *2,696 (25.9)* | *5,468 (52.5)* | *2,250 (21.6)* |
| **Household size** | | | | |
| 0 members | 33,489 | 7,450 (22.2) | 18,267 (54.5) | 7,772 (23.2) |
| 1 member | 52,760 | 23,703 (44.9) | 23,311 (44.2) | 5,746 (10.9) |
| 2-3 members | 30,407 | 10,040 (33.0) | 15,519 (51.0) | 4,848 (15.9) |
| 4-5 members | 7,278 | 2,166 (29.8) | 3,731 (51.3) | 1,381 (19.0) |
| > 5 members | 1,494 | 336 (22.5) | 752 (50.3) | 406 (27.2) |
| *Missing* | *10,294* | *2,660 (25.8)* | *5,421 (52.7)* | *2,213 (21.5)* |
| **Having children** | | | | |
| No | 40,684 | 10,469 (25.7) | 21,669 (53.3) | 8,546 (21.0) |
| Yes | 84,561 | 33,174 (39.2) | 39,823 (47.1) | 11,564 (13.7) |
| *Missing* | *10,477* | *2,712 (25.9)* | *5,509 (52.6)* | *2,256 (21.5)* |
| **Having children under 16** | | | | |
| No | 64,991 | 27,686 (42.6) | 29,572 (45.5) | 7,733 (11.9) |
| Yes | 19,287 | 5,372 (27.9) | 10,119 (52.5) | 3,796 (19.7) |
| *Missing* | *283* | *116 (41.0)* | *132 (46.6)* | *35 (12.4)* |
| **Having disability** | | | | |
| No | 96,817 | 38,373 (39.6) | 46,581 (48.1) | 11,863 (12.3) |
| Prefer not to say | 3,584 | 593 (16.5) | 1,982 (55.3) | 1,009 (28.2) |
| Yes | 24,915 | 4,692 (18.8) | 12,952 (52.0) | 7,271 (29.2) |
| *Missing* | *10,406* | *2,697 (25.9)* | *5,486 (52.7)* | *2,223 (21.4)* |
| **Having long-term conditions** | | | | |
| No | 60,260 | 26,835 (44.5) | 27,894 (46.3) | 5,531 (9.2) |
| Prefer not to say | 3,085 | 685 (22.2) | 1,703 (55.2) | 697 (22.6) |
| Yes | 62,038 | 16,162 (26.1) | 31,956 (51.5) | 13,920 (22.4) |
| *Missing* | *10,339* | *2,673 (25.9)* | *5,448 (52.7)* | *2,218 (21.5)* |

Abbreviations: UCLA, University of California, Los Angeles; DMOL, Direct Measure of Loneliness; ONS, Office for National Statistics; N/n, number of respondents.

**Notes:** Percentages are row percentages and therefore sum to approximately 100% across the three DMOL categories for each respondent subcategory. Findings describe the distribution observed among INTERACT participants and should not be interpreted as population prevalence estimates. The total analytical sample for this table was N=135,722. Missing denotes respondents with no recorded value for the relevant characteristic.

Table S6: Distribution of social-capital categories within respondent characteristics among INTERACT participants

| Category | Subcategory | Low social capital (N=56,887) | | High social capital (N=63,696) | | Total (N=120,583) | |
| --- | --- | --- | --- | --- | --- | --- | --- |
|  |  | N | % | N | % | N | % |
| **Age** | 16-25 | 5192 | 72.0% | 2020 | 28.0% | 7212 | 100.0% |
|  | 26-35 | 7943 | 68.4% | 3673 | 31.6% | 11616 | 100.0% |
|  | 36-45 | 7897 | 58.9% | 5501 | 41.1% | 13398 | 100.0% |
|  | 46-55 | 10322 | 51.9% | 9569 | 48.1% | 19891 | 100.0% |
|  | 56-65 | 13062 | 43.6% | 16900 | 56.4% | 29962 | 100.0% |
|  | >65 | 12435 | 32.4% | 25986 | 67.6% | 38421 | 100.0% |
|  | Missing | 36 | 43.4% | 47 | 56.6% | 83 | 100.0% |
| **Gender** | Female | 35138 | 47.4% | 39063 | 52.6% | 74201 | 100.0% |
|  | Male | 20988 | 46.3% | 24301 | 53.7% | 45289 | 100.0% |
|  | Other | 532 | 74.2% | 185 | 25.8% | 717 | 100.0% |
|  | Would rather not say | 221 | 61.6% | 138 | 38.4% | 359 | 100.0% |
|  | Missing | 8 | 47.1% | 9 | 52.9% | 17 | 100.0% |
| **Education** | A levels/College | 17240 | 48.7% | 18162 | 51.3% | 35402 | 100.0% |
|  | Secondary School | 14548 | 50.8% | 14085 | 49.2% | 28633 | 100.0% |
|  | University Degree or higher | 24928 | 44.3% | 31335 | 55.7% | 56263 | 100.0% |
|  | Missing | 171 | 60.0% | 114 | 40.0% | 285 | 100.0% |
| **Employment** | Employed full-time | 20713 | 55.2% | 16805 | 44.8% | 37518 | 100.0% |
|  | Employed part-time | 6534 | 46.2% | 7622 | 53.8% | 14156 | 100.0% |
|  | Furloughed | 36 | 75.0% | 12.0 | 25.0% | 48.0 | 100.0% |
|  | Other | 2555 | 65.4% | 1350 | 34.6% | 3905 | 100.0% |
|  | Retired | 13928 | 32.7% | 28691 | 67.3% | 42619 | 100.0% |
|  | Self-employed | 3071 | 41.8% | 4277 | 58.2% | 7348 | 100.0% |
|  | Student (full or part-time) | 2579 | 70.0% | 1104 | 30.0% | 3683 | 100.0% |
|  | Unemployed | 5452 | 74.7% | 1843 | 25.3% | 7295 | 100.0% |
|  | Unpaid carer | 971 | 64.8% | 528 | 35.2% | 1499 | 100.0% |
|  | Volunteer (full or part-time) | 547 | 39.0% | 857 | 61.0% | 1404 | 100.0% |
|  | Missing | 501 | 45.2% | 607 | 54.8% | 1108 | 100.0% |
| **Ethnicity** | Asian/Asian British | 2994 | 60.7% | 1935 | 39.3% | 4929 | 100.0% |
|  | British Black/African/Caribbean | 1545 | 72.1% | 598 | 27.9% | 2143 | 100.0% |
|  | Mixed/Multiple ethnic groups | 1207 | 62.1% | 737 | 37.9% | 1944 | 100.0% |
|  | Other ethnic group | 1595 | 62.0% | 978 | 38.0% | 2573 | 100.0% |
|  | White | 48968 | 45.4% | 59004 | 54.6% | 107972 | 100.0% |
|  | White and Black Caribbean | 291 | 65.4% | 154 | 34.6% | 445 | 100.0% |
|  | Missing | 287 | 49.7% | 290 | 50.3% | 577 | 100.0% |
| **Marital Status** | Divorced | 6509 | 53.7% | 5621 | 46.3% | 12130 | 100.0% |
|  | In a relationship | 6733 | 55.5% | 5398 | 44.5% | 12131 | 100.0% |
|  | Married / Civil partnership | 21430 | 36.7% | 36991 | 63.3% | 58421 | 100.0% |
|  | Other | 1719 | 60.3% | 1133 | 39.7% | 2852 | 100.0% |
|  | Widowed | 3343 | 37.4% | 5591 | 62.6% | 8934 | 100.0% |
|  | single | 17099 | 65.7% | 8927 | 34.3% | 26026 | 100.0% |
|  | Missing | 54 | 60.7% | 35 | 39.3% | 89 | 100.0% |
| **Having relatives** | 0 | 4659 | 68.7% | 2123 | 31.3% | 6782 | 100.0% |
|  | 1 | 7354 | 63.7% | 4188 | 36.3% | 11542 | 100.0% |
|  | 2 | 11802 | 58.6% | 8346 | 41.4% | 20148 | 100.0% |
|  | 3 or 4 | 18555 | 48.6% | 19629 | 51.4% | 38184 | 100.0% |
|  | 5-8 | 10189 | 35.6% | 18422 | 64.4% | 28611 | 100.0% |
|  | 9 or more | 4292 | 28.1% | 10964 | 71.9% | 15256 | 100.0% |
|  | Missing | 36 | 60.0% | 24 | 40.0% | 60 | 100.0% |
| **Having friends** | 0 | 8482 | 74.6% | 2887 | 25.4% | 11369 | 100.0% |
|  | 1 | 9084 | 68.5% | 4177 | 31.5% | 13261 | 100.0% |
|  | 2 | 10969 | 59.6% | 7442 | 40.4% | 18411 | 100.0% |
|  | 3 or 4 | 14212 | 46.6% | 16258 | 53.4% | 30470 | 100.0% |
|  | 5-8 | 8293 | 34.1% | 16057 | 65.9% | 24350 | 100.0% |
|  | 9 or more | 5815 | 25.7% | 16844 | 74.3% | 22659 | 100.0% |
|  | Missing | 32 | 50.8% | 31 | 49.2% | 63 | 100.0% |
| **Having pets** | No | 31668 | 47.5% | 35052 | 52.5% | 66720 | 100.0% |
|  | Yes | 25112 | 46.8% | 28544 | 53.2% | 53656 | 100.0% |
|  | Missing | 107 | 51.7% | 100 | 48.3% | 207 | 100.0% |
| **Household size** | 0 members | 16569 | 52.3% | 15087 | 47.7% | 31656 | 100.0% |
|  | 1 member | 20775 | 40.9% | 30009 | 59.1% | 50784 | 100.0% |
|  | 2-3 members | 14721 | 49.8% | 14816 | 50.2% | 29537 | 100.0% |
|  | 4-5 members | 3850 | 54.3% | 3236 | 45.7% | 7086 | 100.0% |
|  | >5 members | 918 | 64.6% | 502 | 35.4% | 1420 | 100.0% |
|  | Missing | 54 | 54.0% | 46 | 46.0% | 100 | 100.0% |
| **Having children** | No | 22627 | 57.4% | 16791 | 42.6% | 39418 | 100.0% |
|  | Yes | 34124 | 42.2% | 46766 | 57.8% | 80890 | 100.0% |
|  | Missing | 136 | 49.5% | 139 | 50.5% | 275 | 100.0% |
| **Having children under 16** | No | 24237 | 39.2% | 37639 | 60.8% | 61876 | 100.0% |
|  | Yes | 9774 | 52.1% | 8976 | 47.9% | 18750 | 100.0% |
|  | Missing | 113 | 42.8% | 151 | 57.2% | 264 | 100.0% |
| **Having disability** | No | 40280 | 43.1% | 53113 | 56.9% | 93393 | 100.0% |
|  | Prefer not to say | 2202 | 66.1% | 1131 | 33.9% | 3333 | 100.0% |
|  | Yes | 14296 | 60.5% | 9350 | 39.5% | 23646 | 100.0% |
|  | Missing | 109 | 51.7% | 102 | 48.3% | 211 | 100.0% |
| **Having long-term conditions** | No | 23010 | 39.7% | 34978 | 60.3% | 57988 | 100.0% |
|  | Prefer not to say | 1714 | 59.6% | 1164 | 40.4% | 2878 | 100.0% |
|  | Yes | 32096 | 53.9% | 27479 | 46.1% | 59575 | 100.0% |
|  | Missing | 67 | 47.2% | 75 | 52.8% | 142 | 100.0% |

Abbreviations: UCLA, University of California, Los Angeles; DMOL, Direct Measure of Loneliness; ONS, Office for National Statistics; N/n, number of respondents.

Notes: Percentages are row percentages and therefore sum to approximately 100% across the low- and high-social-capital categories within each respondent subcategory. Findings describe the distribution observed among INTERACT participants and should not be interpreted as population prevalence estimates. The analytical sample was N=120,583. Missing denotes respondents with no recorded value for the relevant characteristic.

Abbreviations: UCLA, University of California, Los Angeles; DMOL, Direct Measure of Loneliness; SC, social capital; N/n, number of respondents. Percentages describe respondents with observed outcome data and are not population estimates.
