# Supplementary material for: Factors associated with loneliness and social capital in a large volunteer sample of adults in England
Source: BMC Glob Public Health. 2026 Oct 2;4:96. doi: 10.1186/s44263-026-00335-1 (PMC13632327; doi:10.1186/s44263-026-00335-1)
Supplement: Supplementary file 4 [file 44263_2026_335_MOESM4_ESM.docx]

Supplementary material 4: Multiple imputation (MICE) sensitivity analysis

**Methods**

**Missing values in the dataset**

We assessed the distribution of missing data across variables (Fig S1) and calculated the overall proportion of missing values as the number of missing values divided by the total number of values, multiplied by 100. Overall, 6.8% of values were missing across the dataset.

**Multiple Imputation**

To address missing data in our study, we employed multiple imputations using the Multivariate Imputation by Chained Equations (MICE) algorithm. This method was implemented using the ‘mice’ package in R.

**Missing Data Mechanism Assessment**

Prior to imputation, we conducted Little’s test to assess whether the data were Missing Completely At Random (MCAR). The test indicated that the data were not MCAR; multiple imputation was therefore used as a sensitivity analysis alongside the primary complete-case models.

**Imputation Process**

We first examined the missing data pattern using the ‘md.pattern()’ function to understand the structure of missingness in our dataset. Based on this analysis, we tailored our imputation strategy to the specific characteristics of each variable. For the imputation process, we generated five imputed datasets (m =5) using 40 iterations (maxit =40) for each imputation. A seed value of 123 was set to ensure reproducibility of the results.

**Imputation Methods**

We tailored the imputation methods to variable type. Polytomous regression (polyreg) was used for nominal categorical variables, including gender, education, employment, ethnicity, marital status, disability and long-term conditions. Proportional-odds models (polr) were used for ordinal variables, including age group, number of relatives, number of friends and household size. Logistic regression (logreg) was used for binary variables, including pet ownership, having children and the dichotomised social-capital score.

**Diagnostics for the imputation process**

Convergence diagnostics for the multiple imputation process were examined across 40 iterations for the variables in Fig S2. The means and standard deviations demonstrated stable patterns without systematic trends. The consistent patterns and absence of extreme deviations between the five imputed datasets indicated successful convergence of the imputation algorithm and plausible imputed values.

Fig S1: Missing Data Distribution Across Variables in Dataset. This figure displays the number of missing values (#Missing) for various demographic and social variables in a dataset. The variables are arranged vertically, with the Social Capital Scale showing the highest number of missing entries, followed by variables like employment, ethnicity and education.

Fig S2: Convergence diagnostics for multiple imputation. Trace plots showing means (left panels) and standard deviations (right panels) across 40 iterations for all imputed variables. Each coloured line represents one of the five imputed datasets. The plots demonstrate the convergence patterns for demographic variables (age group, gender, education), social variables (employment, ethnicity, marital status, relatives, friends), household characteristics (pets, household, children), health-related variables (disability, long-term conditions) and outcome measures (UCLA, DMOL, Social capital scale).

**Post-Imputation Analysis**

Following imputation, the regression models were fitted separately in each of the five imputed datasets and pooled using Rubin’s rules to incorporate within- and between-imputation variability (Tables S7–S9). Estimates are presented as odds ratios with 95% model-based intervals; p-values were not used for interpretation.

**Association between demographic and social factors and loneliness as measured by the UCLA Loneliness Scale**

Pooled estimates from the imputed datasets were broadly consistent with the complete-case UCLA-3 model (Table S7). Older age was associated with lower odds of membership in a more severe loneliness category; the adjusted odds ratio for respondents aged over 65 years was (aOR = 0.15, 95% MBI: 0.14–0.16) relative to respondents aged 16–25 years. Males also had lower adjusted odds than females (aOR = 0.81, 95% MBI: 0.79–0.83), while the adjusted estimate for the ‘Other’ gender category was attenuated relative to the unadjusted estimate.

Educational attainment showed a change in direction after adjustment: university graduates had higher adjusted odds of more severe UCLA-3 loneliness than respondents with secondary-school education (aOR = 1.21, 95% MBI: 1.17–1.24). Relative to respondents in full-time employment, unemployed respondents had higher adjusted odds (aOR = 1.79, 95% MBI: 1.68–1.91). Relative to single respondents, married or civil-partnered respondents had lower adjusted odds (aOR = 0.44, 95% MBI: 0.42–0.46), whereas widowed respondents had higher adjusted odds (aOR = 1.34, 95% MBI: 1.27–1.42).

Greater social contact was associated with lower UCLA-3 loneliness. Relative to respondents reporting no friends, those reporting nine or more friends had substantially lower adjusted odds (aOR = 0.09, 95% MBI: 0.09–0.10). Respondents with a disability (aOR = 1.77, 95% MBI: 1.71–1.82) or long-term condition (aOR = 1.67, 95% MBI: 1.62–1.72) had higher adjusted odds than the respective reference groups.

Table S7: Association between demographic and social factors and loneliness measured using the UCLA-3

| Characteristic | Unadjusted estimate | Adjusted estimate |
| --- | --- | --- |
| Age |  |  |
| 16-25 | Ref. | Ref. |
| 26-35 | (OR = 0.70, 95% MBI: 0.67–0.74) | (aOR = 0.66, 95% MBI: 0.62–0.71) |
| 36-45 | (OR = 0.59, 95% MBI: 0.56–0.62) | (aOR = 0.50, 95% MBI: 0.46–0.53) |
| 46-55 | (OR = 0.43, 95% MBI: 0.41–0.45) | (aOR = 0.33, 95% MBI: 0.31–0.36) |
| 56-65 | (OR = 0.27, 95% MBI: 0.25–0.28) | (aOR = 0.23, 95% MBI: 0.21–0.25) |
| >65 | (OR = 0.15, 95% MBI: 0.15–0.16) | (aOR = 0.15, 95% MBI: 0.14–0.16) |
| Gender |  |  |
| Female | Ref. | Ref. |
| Male | (OR = 0.79, 95% MBI: 0.77–0.80) | (aOR = 0.81, 95% MBI: 0.79–0.83) |
| Other | (OR = 3.37, 95% MBI: 2.93–3.87) | (aOR = 1.19, 95% MBI: 1.02–1.40) |
| Would rather not say | (OR = 1.98, 95% MBI: 1.67–2.34) | (aOR = 0.99, 95% MBI: 0.81–1.20) |
| Education |  |  |
| Secondary school | Ref. | Ref. |
| A levels/College | (OR = 1.03, 95% MBI: 1.00–1.05) | (aOR = 1.10, 95% MBI: 1.07–1.14) |
| University Degree or higher | (OR = 0.79, 95% MBI: 0.77–0.81) | (aOR = 1.21, 95% MBI: 1.17–1.24) |
| Employment |  |  |
| Employed full-time | Ref. | Ref. |
| Employed part-time | (OR = 0.80, 95% MBI: 0.77–0.83) | (aOR = 1.06, 95% MBI: 1.02–1.11) |
| Furloughed | (OR = 2.35, 95% MBI: 1.54–3.59) | (aOR = 2.28, 95% MBI: 1.37–3.79) |
| Volunteer (full or part-time) | (OR = 0.61, 95% MBI: 0.55–0.67) | (aOR = 1.09, 95% MBI: 0.98–1.21) |
| Retired | (OR = 0.42, 95% MBI: 0.41–0.43) | (aOR = 0.94, 95% MBI: 0.90–0.98) |
| Self-employed | (OR = 0.69, 95% MBI: 0.66–0.72) | (aOR = 1.11, 95% MBI: 1.06–1.17) |
| Student (full or part-time) | (OR = 2.20, 95% MBI: 2.07–2.34) | (aOR = 1.26, 95% MBI: 1.16–1.36) |
| Unemployed | (OR = 4.67, 95% MBI: 4.44–4.92) | (aOR = 1.79, 95% MBI: 1.68–1.91) |
| Unpaid carer | (OR = 2.58, 95% MBI: 2.33–2.85) | (aOR = 2.05, 95% MBI: 1.84–2.29) |
| Other | (OR = 3.31, 95% MBI: 3.10–3.53) | (aOR = 1.71, 95% MBI: 1.59–1.84) |
| Ethnicity |  |  |
| White | Ref. | Ref. |
| British Black/African/Caribbean | (OR = 1.72, 95% MBI: 1.59–1.85) | (aOR = 1.06, 95% MBI: 0.96–1.16) |
| Mixed/Multiple ethnic groups | (OR = 1.83, 95% MBI: 1.68–1.99) | (aOR = 1.04, 95% MBI: 0.95–1.15) |
| White and Black Caribbean | (OR = 2.34, 95% MBI: 1.95–2.81) | (aOR = 1.16, 95% MBI: 0.94–1.43) |
| Asian/Asian British | (OR = 1.76, 95% MBI: 1.66–1.87) | (aOR = 1.22, 95% MBI: 1.14–1.31) |
| Other ethnic group | (OR = 1.60, 95% MBI: 1.49–1.72) | (aOR = 1.04, 95% MBI: 0.96–1.13) |
| Marital status |  |  |
| single | Ref. | Ref. |
| Divorced | (OR = 0.62, 95% MBI: 0.59–0.64) | (aOR = 1.05, 95% MBI: 1.00–1.10) |
| In a relationship | (OR = 0.42, 95% MBI: 0.40–0.44) | (aOR = 0.51, 95% MBI: 0.49–0.54) |
| Married / Civil partnership | (OR = 0.18, 95% MBI: 0.18–0.19) | (aOR = 0.44, 95% MBI: 0.42–0.46) |
| Widowed | (OR = 0.45, 95% MBI: 0.43–0.47) | (aOR = 1.34, 95% MBI: 1.27–1.42) |
| Other | (OR = 0.76, 95% MBI: 0.71–0.81) | (aOR = 0.98, 95% MBI: 0.91–1.06) |
| Number of relatives |  |  |
| 0 relatives | Ref. | Ref. |
| 1 relative | (OR = 0.91, 95% MBI: 0.86–0.97) | (aOR = 1.02, 95% MBI: 0.96–1.08) |
| 2 relatives | (OR = 0.70, 95% MBI: 0.66–0.73) | (aOR = 0.85, 95% MBI: 0.80–0.90) |
| 3 or 4 relatives | (OR = 0.36, 95% MBI: 0.34–0.38) | (aOR = 0.57, 95% MBI: 0.54–0.61) |
| 5-8 relatives | (OR = 0.16, 95% MBI: 0.15–0.17) | (aOR = 0.39, 95% MBI: 0.37–0.42) |
| 9 or more relatives | (OR = 0.09, 95% MBI: 0.08–0.09) | (aOR = 0.29, 95% MBI: 0.27–0.31) |
| Number of friends |  |  |
| 0 friends | Ref. | Ref. |
| 1 friend | (OR = 0.78, 95% MBI: 0.74–0.82) | (aOR = 0.79, 95% MBI: 0.75–0.83) |
| 2 friends | (OR = 0.46, 95% MBI: 0.44–0.48) | (aOR = 0.51, 95% MBI: 0.49–0.54) |
| 3 or 4 friends | (OR = 0.22, 95% MBI: 0.21–0.23) | (aOR = 0.29, 95% MBI: 0.27–0.30) |
| 5-8 friends | (OR = 0.10, 95% MBI: 0.10–0.11) | (aOR = 0.16, 95% MBI: 0.15–0.17) |
| 9 or more friends | (OR = 0.05, 95% MBI: 0.05–0.05) | (aOR = 0.09, 95% MBI: 0.09–0.10) |
| Having pet |  |  |
| No | Ref. | Ref. |
| Yes | (OR = 1.25, 95% MBI: 1.22–1.27) | (aOR = 1.05, 95% MBI: 1.02–1.07) |
| Number of household members |  |  |
| 0 members | Ref. | Ref. |
| 1 member | (OR = 0.39, 95% MBI: 0.38–0.40) | (aOR = 0.74, 95% MBI: 0.71–0.78) |
| 2-3 members | (OR = 0.64, 95% MBI: 0.62–0.66) | (aOR = 0.70, 95% MBI: 0.66–0.73) |
| 4-5 members | (OR = 0.73, 95% MBI: 0.69–0.76) | (aOR = 0.66, 95% MBI: 0.62–0.71) |
| > 5 members | (OR = 1.06, 95% MBI: 0.96–1.17) | (aOR = 0.67, 95% MBI: 0.60–0.76) |
| Children |  |  |
| No | Ref. | Ref. |
| Yes | (OR = 0.48, 95% MBI: 0.47–0.49) | (aOR = 1.06, 95% MBI: 1.03–1.10) |
| Disability |  |  |
| No | Ref. | Ref. |
| Yes | (OR = 3.51, 95% MBI: 3.41–3.60) | (aOR = 1.77, 95% MBI: 1.71–1.82) |
| Would rather not say | (OR = 3.48, 95% MBI: 3.27–3.71) | (aOR = 1.56, 95% MBI: 1.45–1.68) |
| Longterm conditions |  |  |
| No | Ref. | Ref. |
| Yes | (OR = 2.76, 95% MBI: 2.70–2.83) | (aOR = 1.67, 95% MBI: 1.62–1.72) |
| Would rather not say | (OR = 3.03, 95% MBI: 2.82–3.26) | (aOR = 1.66, 95% MBI: 1.54–1.80) |
| ¶: Crude ordinal logistic regression model. †: Ordinal logistic regression model adjusted for age, gender, education, employment, ethnicity, marital status, number of relatives, number of friends, pet ownership, household size, children, disability and long-term conditions. Intervals are 95% model-based intervals pooled using Rubin’s rules and are conditional on the imputation and regression models; they do not address uncertainty or bias arising from non-probability selection. Number of observations in each imputed dataset = 135,725. | | |

Abbreviations: UCLA, University of California, Los Angeles; DMOL, Direct Measure of Loneliness; ONS, Office for National Statistics; N/n, number of respondents. OR, odds ratio; aOR, adjusted odds ratio; MBI, model-based interval; Ref., reference category.

Fig S3: Forest plot of associations between demographic and social factors and loneliness measured using the UCLA-3

**Association between demographic and social factors and loneliness measured using the Direct Measure of Loneliness (DMOL)**

Pooled estimates from the imputed datasets were broadly consistent with the complete-case DMOL model (Table S8). Older age was associated with lower odds of membership in a more severe DMOL category; the adjusted odds ratio for respondents aged over 65 years was (aOR = 0.19, 95% MBI: 0.18–0.21) relative to respondents aged 16–25 years. Males also had lower adjusted odds than females (aOR = 0.74, 95% MBI: 0.73–0.76), while the adjusted estimate for the ‘Other’ gender category was close to the null.

University graduates had slightly lower adjusted odds of more severe DMOL loneliness than respondents with secondary-school education (aOR = 0.95, 95% MBI: 0.92–0.98). Relative to respondents in full-time employment, unemployed respondents had higher adjusted odds (aOR = 1.67, 95% MBI: 1.57–1.78). Relative to single respondents, married or civil-partnered respondents had lower adjusted odds (aOR = 0.45, 95% MBI: 0.43–0.47), whereas widowed respondents had higher adjusted odds (aOR = 1.48, 95% MBI: 1.40–1.56).

Greater social contact was associated with lower DMOL loneliness. Relative to respondents reporting no friends, those reporting nine or more friends had lower adjusted odds (aOR = 0.16, 95% MBI: 0.15–0.17). Respondents with a disability (aOR = 1.44, 95% MBI: 1.39–1.48) or long-term condition (aOR = 1.54, 95% MBI: 1.50–1.59) had higher adjusted odds than the respective reference groups.

Table S8: Association between demographic and social factors and loneliness measured using the DMOL

| Characteristic | Unadjusted estimate | Adjusted estimate |
| --- | --- | --- |
| Age |  |  |
| 16-25 | Ref. | Ref. |
| 26-35 | (OR = 0.76, 95% MBI: 0.72–0.80) | (aOR = 0.74, 95% MBI: 0.69–0.79) |
| 36-45 | (OR = 0.64, 95% MBI: 0.61–0.67) | (aOR = 0.55, 95% MBI: 0.52–0.59) |
| 46-55 | (OR = 0.49, 95% MBI: 0.46–0.51) | (aOR = 0.39, 95% MBI: 0.36–0.42) |
| 56-65 | (OR = 0.31, 95% MBI: 0.30–0.32) | (aOR = 0.27, 95% MBI: 0.26–0.29) |
| >65 | (OR = 0.20, 95% MBI: 0.19–0.21) | (aOR = 0.19, 95% MBI: 0.18–0.21) |
| Gender |  |  |
| Female | Ref | Ref. |
| Male | (OR = 0.73, 95% MBI: 0.72–0.75) | (aOR = 0.74, 95% MBI: 0.73–0.76) |
| Other | (OR = 2.42, 95% MBI: 2.12–2.76) | (aOR = 0.95, 95% MBI: 0.82–1.10) |
| Would rather not say | (OR = 1.73, 95% MBI: 1.46–2.04) | (aOR = 0.89, 95% MBI: 0.74–1.07) |
| Education |  |  |
| Secondary school | Ref | Ref. |
| A levels/College | (OR = 0.93, 95% MBI: 0.91–0.96) | (aOR = 0.96, 95% MBI: 0.93–0.99) |
| University Degree or higher | (OR = 0.70, 95% MBI: 0.68–0.72) | (aOR = 0.95, 95% MBI: 0.92–0.98) |
| Employment |  |  |
| Employed full-time | Ref | Ref. |
| Employed part-time | (OR = 0.87, 95% MBI: 0.84–0.90) | (aOR = 1.07, 95% MBI: 1.03–1.12) |
| Furloughed | (OR = 1.96, 95% MBI: 1.29–2.98) | (aOR = 1.51, 95% MBI: 0.96–2.38) |
| Volunteer (full or part-time) | (OR = 0.64, 95% MBI: 0.58–0.71) | (aOR = 1.03, 95% MBI: 0.91–1.15) |
| Retired | (OR = 0.49, 95% MBI: 0.48–0.50) | (aOR = 0.95, 95% MBI: 0.91–0.99) |
| Self-employed | (OR = 0.72, 95% MBI: 0.69–0.75) | (aOR = 1.09, 95% MBI: 1.04–1.15) |
| Student (full or part-time) | (OR = 1.94, 95% MBI: 1.83–2.06) | (aOR = 1.08, 95% MBI: 1.00–1.17) |
| Unemployed | (OR = 4.09, 95% MBI: 3.89–4.31) | (aOR = 1.67, 95% MBI: 1.57–1.78) |
| Unpaid carer | (OR = 2.15, 95% MBI: 1.94–2.37) | (aOR = 1.58, 95% MBI: 1.42–1.75) |
| Other | (OR = 2.93, 95% MBI: 2.74–3.14) | (aOR = 1.56, 95% MBI: 1.45–1.68) |
| Ethnicity |  |  |
| White | Ref | Ref. |
| British Black/African/Caribbean | (OR = 1.80, 95% MBI: 1.67–1.95) | (aOR = 1.18, 95% MBI: 1.08–1.29) |
| Mixed/Multiple ethnic groups | (OR = 1.67, 95% MBI: 1.54–1.82) | (aOR = 1.04, 95% MBI: 0.94–1.14) |
| White and Black Caribbean | (OR = 2.20, 95% MBI: 1.82–2.67) | (aOR = 1.11, 95% MBI: 0.91–1.35) |
| Asian/Asian British | (OR = 1.85, 95% MBI: 1.75–1.96) | (aOR = 1.39, 95% MBI: 1.31–1.48) |
| Other ethnic group | (OR = 1.73, 95% MBI: 1.61–1.87) | (aOR = 1.27, 95% MBI: 1.17–1.38) |
| Marital status |  |  |
| single | Ref | Ref. |
| Divorced | (OR = 0.65, 95% MBI: 0.63–0.68) | (aOR = 1.00, 95% MBI: 0.95–1.05) |
| In a relationship | (OR = 0.40, 95% MBI: 0.38–0.42) | (aOR = 0.48, 95% MBI: 0.46–0.51) |
| Married / Civil partnership | (OR = 0.21, 95% MBI: 0.20–0.21) | (aOR = 0.45, 95% MBI: 0.43–0.47) |
| Widowed | (OR = 0.58, 95% MBI: 0.55–0.60) | (aOR = 1.48, 95% MBI: 1.40–1.56) |
| Other | (OR = 0.82, 95% MBI: 0.77–0.89) | (aOR = 1.01, 95% MBI: 0.94–1.10) |
| Number of relatives |  |  |
| 0 relatives | Ref | Ref. |
| 1 relative | (OR = 0.88, 95% MBI: 0.83–0.93) | (aOR = 0.95, 95% MBI: 0.90–1.01) |
| 2 relatives | (OR = 0.70, 95% MBI: 0.66–0.73) | (aOR = 0.82, 95% MBI: 0.77–0.87) |
| 3 or 4 relatives | (OR = 0.40, 95% MBI: 0.38–0.42) | (aOR = 0.60, 95% MBI: 0.57–0.63) |
| 5-8 relatives | (OR = 0.19, 95% MBI: 0.18–0.20) | (aOR = 0.41, 95% MBI: 0.39–0.44) |
| 9 or more relatives | (OR = 0.11, 95% MBI: 0.10–0.12) | (aOR = 0.30, 95% MBI: 0.28–0.32) |
| Number of friends |  |  |
| 0 friends | Ref | Ref. |
| 1 friend | (OR = 0.81, 95% MBI: 0.77–0.85) | (aOR = 0.83, 95% MBI: 0.79–0.87) |
| 2 friends | (OR = 0.53, 95% MBI: 0.50–0.55) | (aOR = 0.61, 95% MBI: 0.59–0.64) |
| 3 or 4 friends | (OR = 0.29, 95% MBI: 0.28–0.30) | (aOR = 0.40, 95% MBI: 0.39–0.42) |
| 5-8 friends | (OR = 0.15, 95% MBI: 0.15–0.16) | (aOR = 0.26, 95% MBI: 0.25–0.27) |
| 9 or more friends | (OR = 0.08, 95% MBI: 0.08–0.08) | (aOR = 0.16, 95% MBI: 0.15–0.17) |
| Having pet |  |  |
| No | Ref | Ref. |
| Yes | (OR = 1.16, 95% MBI: 1.13–1.18) | (aOR = 0.99, 95% MBI: 0.97–1.02) |
| Number of household members |  |  |
| 0 members | Ref | Ref. |
| 1 member | (OR = 0.40, 95% MBI: 0.38–0.41) | (aOR = 0.78, 95% MBI: 0.74–0.81) |
| 2-3 members | (OR = 0.62, 95% MBI: 0.60–0.64) | (aOR = 0.73, 95% MBI: 0.70–0.76) |
| 4-5 members | (OR = 0.73, 95% MBI: 0.70–0.77) | (aOR = 0.75, 95% MBI: 0.70–0.80) |
| > 5 members | (OR = 1.07, 95% MBI: 0.97–1.18) | (aOR = 0.80, 95% MBI: 0.71–0.89) |
| Children |  |  |
| No | Ref | Ref. |
| Yes | (OR = 0.56, 95% MBI: 0.55–0.57) | (aOR = 1.20, 95% MBI: 1.16–1.24) |
| Disability |  |  |
| No | Ref | Ref. |
| Yes | (OR = 2.88, 95% MBI: 2.80–2.96) | (aOR = 1.44, 95% MBI: 1.39–1.48) |
| Would rather not say | (OR = 2.95, 95% MBI: 2.77–3.14) | (aOR = 1.33, 95% MBI: 1.24–1.42) |
| Longterm conditions |  |  |
| No | Ref | Ref. |
| Yes | (OR = 2.44, 95% MBI: 2.38–2.49) | (aOR = 1.54, 95% MBI: 1.50–1.59) |
| Would rather not say | (OR = 2.7, 95% MBI: 2.51–2.89) | (aOR = 1.52, 95% MBI: 1.41–1.63) |
| ¶: Crude ordinal logistic regression model. †: Ordinal logistic regression model adjusted for age, gender, education, employment, ethnicity, marital status, number of relatives, number of friends, pet ownership, household size, children, disability and long-term conditions. Intervals are 95% model-based intervals pooled using Rubin’s rules and are conditional on the imputation and regression models; they do not address uncertainty or bias arising from non-probability selection. Number of observations in each imputed dataset = 135,725. | | |

Abbreviations: UCLA, University of California, Los Angeles; DMOL, Direct Measure of Loneliness; ONS, Office for National Statistics; N/n, number of respondents. OR, odds ratio; aOR, adjusted odds ratio; MBI, model-based interval; Ref., reference category.

Fig S4: Forest plot of associations between demographic and social factors and loneliness measured using the DMOL

**Association between demographic and social factors and the Social Capital Scale**

Pooled estimates from the imputed datasets were broadly consistent with the complete-case social-capital model (Table S9). Relative to respondents aged 16–25 years, respondents aged over 65 years had higher adjusted odds of high social capital (aOR = 3.24, 95% MBI: 2.97–3.53). Males had slightly higher adjusted odds than females (aOR = 1.04, 95% MBI: 1.01–1.06), whereas respondents in the ‘Other’ gender category had lower adjusted odds (aOR = 0.73, 95% MBI: 0.61–0.88).

University graduates had higher adjusted odds of high social capital than respondents with secondary-school education (aOR = 1.15, 95% MBI: 1.11–1.19). Relative to respondents in full-time employment, retired respondents had higher adjusted odds (aOR = 1.48, 95% MBI: 1.41–1.56), whereas unemployed respondents had lower adjusted odds (aOR = 0.90, 95% MBI: 0.85–0.96). Relative to single respondents, married or civil-partnered respondents had higher adjusted odds (aOR = 1.59, 95% MBI: 1.51–1.66).

Greater social contact was associated with higher social capital. Relative to respondents reporting no friends, those reporting nine or more friends had higher adjusted odds of high social capital (aOR = 5.20, 95% MBI: 4.91–5.51). Respondents with a disability (aOR = 0.77, 95% MBI: 0.74–0.80) or long-term condition (aOR = 0.82, 95% MBI: 0.80–0.84) had lower adjusted odds than the respective reference groups.

Table S9: Association between demographic and social factors and the Social Capital Scale

| Characteristic | Unadjusted estimate | Adjusted estimate |
| --- | --- | --- |
| Age |  |  |
| 16-25 | Ref. | Ref. |
| 26-35 | (OR = 1.18, 95% MBI: 1.11–1.26) | (aOR = 1.16, 95% MBI: 1.07–1.25) |
| 36-45 | (OR = 1.77, 95% MBI: 1.67–1.88) | (aOR = 1.81, 95% MBI: 1.68–1.95) |
| 46-55 | (OR = 2.34, 95% MBI: 2.21–2.47) | (aOR = 2.39, 95% MBI: 2.21–2.58) |
| 56-65 | (OR = 3.24, 95% MBI: 3.07–3.42) | (aOR = 2.72, 95% MBI: 2.52–2.94) |
| >65 | (OR = 5.25, 95% MBI: 4.97–5.54) | (aOR = 3.24, 95% MBI: 2.97–3.53) |
| Gender |  |  |
| Female | Ref. | Ref. |
| Male | (OR = 1.03, 95% MBI: 1.01–1.06) | (aOR = 1.04, 95% MBI: 1.01–1.06) |
| Other | (OR = 0.33, 95% MBI: 0.28–0.39) | (aOR = 0.73, 95% MBI: 0.61–0.88) |
| Would rather not say | (OR = 0.57, 95% MBI: 0.45–0.72) | (aOR = 1.09, 95% MBI: 0.82–1.46) |
| Education |  |  |
| Secondary school | Ref. | Ref. |
| A levels/College | (OR = 1.09, 95% MBI: 1.05–1.12) | (aOR = 1.14, 95% MBI: 1.10–1.18) |
| University Degree or higher | (OR = 1.29, 95% MBI: 1.26–1.33) | (aOR = 1.15, 95% MBI: 1.11–1.19) |
| Employment |  |  |
| Employed full-time | Ref. | Ref. |
| Employed part-time | (OR = 1.43, 95% MBI: 1.38–1.49) | (aOR = 1.18, 95% MBI: 1.13–1.23) |
| Furloughed | (OR = 0.78, 95% MBI: 0.51–1.20) | (aOR = 0.99, 95% MBI: 0.61–1.60) |
| Volunteer (full or part-time) | (OR = 1.91, 95% MBI: 1.72–2.12) | (aOR = 1.27, 95% MBI: 1.12–1.43) |
| Retired | (OR = 2.51, 95% MBI: 2.43–2.60) | (aOR = 1.48, 95% MBI: 1.41–1.56) |
| Self-employed | (OR = 1.71, 95% MBI: 1.62–1.81) | (aOR = 1.26, 95% MBI: 1.18–1.33) |
| Student (full or part-time) | (OR = 0.54, 95% MBI: 0.51–0.59) | (aOR = 0.92, 95% MBI: 0.83–1.01) |
| Unemployed | (OR = 0.42, 95% MBI: 0.39–0.44) | (aOR = 0.90, 95% MBI: 0.85–0.96) |
| Unpaid carer | (OR = 0.68, 95% MBI: 0.61–0.77) | (aOR = 0.88, 95% MBI: 0.77–1.00) |
| Other | (OR = 0.66, 95% MBI: 0.61–0.71) | (aOR = 1.08, 95% MBI: 0.99–1.17) |
| Ethnicity |  |  |
| White | Ref. | Ref. |
| British Black/African/Caribbean | (OR = 0.32, 95% MBI: 0.29–0.36) | (aOR = 0.48, 95% MBI: 0.43–0.53) |
| Mixed/Multiple ethnic groups | (OR = 0.50, 95% MBI: 0.46–0.56) | (aOR = 0.79, 95% MBI: 0.71–0.89) |
| White and Black Caribbean | (OR = 0.43, 95% MBI: 0.35–0.53) | (aOR = 0.76, 95% MBI: 0.60–0.95) |
| Asian/Asian British | (OR = 0.54, 95% MBI: 0.51–0.58) | (aOR = 0.83, 95% MBI: 0.77–0.89) |
| Other ethnic group | (OR = 0.51, 95% MBI: 0.46–0.55) | (aOR = 0.68, 95% MBI: 0.62–0.74) |
| Marital status |  |  |
| single | Ref. | Ref. |
| Divorced | (OR = 1.66, 95% MBI: 1.59–1.74) | (aOR = 1.03, 95% MBI: 0.98–1.09) |
| In a relationship | (OR = 1.52, 95% MBI: 1.45–1.59) | (aOR = 1.21, 95% MBI: 1.15–1.28) |
| Married / Civil partnership | (OR = 3.28, 95% MBI: 3.17–3.38) | (aOR = 1.59, 95% MBI: 1.51–1.66) |
| Widowed | (OR = 3.20, 95% MBI: 3.05–3.35) | (aOR = 1.39, 95% MBI: 1.32–1.48) |
| Other | (OR = 1.26, 95% MBI: 1.16–1.37) | (aOR = 1.00, 95% MBI: 0.92–1.09) |
| Number of relatives |  |  |
| 0 relatives | Ref. | Ref. |
| 1 relative | (OR = 1.25, 95% MBI: 1.17–1.33) | (aOR = 1.16, 95% MBI: 1.08–1.25) |
| 2 relatives | (OR = 1.56, 95% MBI: 1.46–1.66) | (aOR = 1.37, 95% MBI: 1.28–1.47) |
| 3 or 4 relatives | (OR = 2.33, 95% MBI: 2.20–2.47) | (aOR = 1.67, 95% MBI: 1.56–1.79) |
| 5-8 relatives | (OR = 3.96, 95% MBI: 3.74–4.20) | (aOR = 2.06, 95% MBI: 1.93–2.21) |
| 9 or more relatives | (OR = 5.57, 95% MBI: 5.23–5.93) | (aOR = 2.31, 95% MBI: 2.14–2.49) |
| Number of friends |  |  |
| 0 friends | Ref. | Ref. |
| 1 friend | (OR = 1.35, 95% MBI: 1.28–1.43) | (aOR = 1.33, 95% MBI: 1.25–1.40) |
| 2 friends | (OR = 1.99, 95% MBI: 1.89–2.09) | (aOR = 1.78, 95% MBI: 1.68–1.87) |
| 3 or 4 friends | (OR = 3.35, 95% MBI: 3.19–3.51) | (aOR = 2.62, 95% MBI: 2.49–2.77) |
| 5-8 friends | (OR = 5.64, 95% MBI: 5.35–5.94) | (aOR = 3.82, 95% MBI: 3.61–4.05) |
| 9 or more friends | (OR = 8.50, 95% MBI: 8.08–8.94) | (aOR = 5.20, 95% MBI: 4.91–5.51) |
| Having pet |  |  |
| No | Ref. | Ref. |
| Yes | (OR = 1.03, 95% MBI: 1.01–1.05) | (aOR = 1.24, 95% MBI: 1.21–1.27) |
| Number of household members |  |  |
| 0 members | Ref. | Ref. |
| 1 member | (OR = 1.52, 95% MBI: 1.48–1.57) | (aOR = 1.05, 95% MBI: 1.01–1.10) |
| 2-3 members | (OR = 1.09, 95% MBI: 1.05–1.12) | (aOR = 1.13, 95% MBI: 1.08–1.19) |
| 4-5 members | (OR = 0.91, 95% MBI: 0.87–0.96) | (aOR = 1.09, 95% MBI: 1.02–1.17) |
| > 5 members | (OR = 0.61, 95% MBI: 0.55–0.69) | (aOR = 0.99, 95% MBI: 0.88–1.13) |
| Children |  |  |
| No | Ref. | Ref. |
| Yes | (OR = 1.84, 95% MBI: 1.80–1.89) | (aOR = 0.93, 95% MBI: 0.90–0.96) |
| Disability |  |  |
| No | Ref. | Ref. |
| Yes | (OR = 0.50, 95% MBI: 0.49–0.51) | (aOR = 0.77, 95% MBI: 0.74–0.80) |
| Would rather not say | (OR = 0.39, 95% MBI: 0.36–0.42) | (aOR = 0.68, 95% MBI: 0.63–0.74) |
| Longterm conditions |  |  |
| No | Ref. | Ref. |
| Yes | (OR = 0.57, 95% MBI: 0.55–0.58) | (aOR = 0.82, 95% MBI: 0.80–0.84) |
| Would rather not say | (OR = 0.45, 95% MBI: 0.42–0.49) | (aOR = 0.74, 95% MBI: 0.68–0.82) |
| ¶: Crude binary logistic regression model. †: Binary logistic regression model adjusted for age, gender, education, employment, ethnicity, marital status, number of relatives, number of friends, pet ownership, household size, children, disability and long-term conditions. Intervals are 95% model-based intervals pooled using Rubin’s rules and are conditional on the imputation and regression models; they do not address uncertainty or bias arising from non-probability selection. Number of observations in each imputed dataset = 135,725. | | |

Abbreviations: UCLA, University of California, Los Angeles; DMOL, Direct Measure of Loneliness; ONS, Office for National Statistics; N/n, number of respondents. OR, odds ratio; aOR, adjusted odds ratio; MBI, model-based interval; Ref., reference category.

Fig S5: Forest plot of associations between demographic and social factors and social capital
