# Supplementary material for: Factors associated with loneliness and social capital in a large volunteer sample of adults in England
Source: BMC Glob Public Health. 2026 Oct 2;4:96. doi: 10.1186/s44263-026-00335-1 (PMC13632327; doi:10.1186/s44263-026-00335-1)
Supplement: Supplementary file 5 [file 44263_2026_335_MOESM5_ESM.docx]

Supplementary material 5: STROBE checklist

Table S10: Cross-sectional reporting checklist for the revised INTERACT analysis. Entries identify what is reported and disclose gaps; they do not certify unreported procedures.

| **Item / reporting domain** | **Manuscript location and reporting status** |
| --- | --- |
| 1a–b \| Design and abstract | Abstract: Methods states cross-sectional analysis; Background, Results and Conclusions summarise the purpose, findings and non-probability sampling limitation. |
| 2 \| Rationale | Background: rationale for adjusted analyses of loneliness and social capital. |
| 3 \| Objectives | Background, final paragraph: primary and secondary aims; exploratory hypothesis generation. |
| 4 \| Design | Methods, opening paragraph and Study Design and Data Source. |
| 5 \| Setting and dates | England; recruitment sources are described in Participants and Sampling. Study Design and Data Source states that responses were collected between 1 March and 31 July 2023. |
| 6a \| Eligibility and recruitment | Methods, Participants and Sampling: adults aged ≥16 years and recruitment routes. Detailed eligibility/exclusion criteria are referred to reference 6. |
| 7 \| Variables | Methods, Measures and Statistical Analysis; Supplementary material 2: Tables S1–S3; model covariates in Tables 1–3. |
| 8 \| Measurement | Self-reported instruments and scoring: Measures; original survey in Supplementary material 1; full item definitions in Supplementary material 2. |
| 9 \| Bias | Methods distinguishes within-sample estimates from population inference. Discussion addresses selection, measurement, confounding and missing contextual variables. |
| 10 \| Sample size | Methods reports 135,725 available respondents and outcome-specific complete-case analysis. No analysis-specific prospective sample-size calculation is reported. |
| 11 \| Numeric variables | Measures describes UCLA-3 score categories and the social-capital threshold; Tables 1–3 show covariate boundaries. |
| 12a \| Adjustment | Statistical Analysis describes cumulative-logit and binary logistic models, covariate rationale and interval interpretation. |
| 12b \| Subgroups/interactions | Discussion explicitly states that main-effects models were used and interactions were not included. |
| 12c \| Missingness | Handling of missing data; Supplementary material 4 describes five imputations, 40 iterations and pooling. |
| 12d \| Sampling strategy | Statistical Analysis: non-probability sampling, unknown inclusion probabilities and no survey weighting. |
| 12e \| Sensitivity analyses | Supplementary material 4: Figs S1–S5 and Tables S7–S9. |
| 13a–c \| Participant accounting | Methods/Results provide total and outcome/model counts. Numbers invited, eligibility exclusions and reasons for non-participation are not supplied; a complete recruitment flow cannot be reconstructed. |
| 14a \| Characteristics | Supplementary material 3: Tables S4–S6 provide respondent characteristics and outcome distributions. |
| 14b \| Missing-data counts | Supplementary material 3 includes missing covariate categories; Supplementary material 4: Fig S1 displays missingness. Item-level missingness is not fully tabulated in the main manuscript. |
| 15 \| Outcome summaries | Results and Supplementary material 3: Tables S4–S6. |
| 16a \| Estimates and precision | Tables 1–3 and Results report unadjusted/adjusted estimates and model-based intervals; these are explicitly not population confidence intervals. |
| 16b \| Category boundaries | Measures and Tables 1–3. |
| 16c \| Absolute risk | Not applicable: no longitudinal risk or causal risk estimates are reported. |
| 17 \| Additional analyses | Multiple-imputation sensitivity analysis in Supplementary material 4. |
| 18 \| Main findings | Discussion, opening paragraphs. |
| 19 \| Limitations | Discussion covers volunteer selection, self-report, outcome categorisation, omitted contextual variables and main-effects modelling. |
| 20 \| Interpretation | Discussion and Conclusions retain cautious, sample-conditional, hypothesis-generating interpretation. |
| 21 \| Transferability | Discussion and Conclusions explicitly reject population-representative interpretation. |
| 22 \| Funding | Declarations: Funding states “Not applicable.” NIHR support acknowledgement is separate. |

STROBE: Strengthening the Reporting of Observational Studies in Epidemiology. Item labels are condensed; consult the original checklist for full wording. Source: von Elm et al. PLoS Medicine. 2007;4:e296. https://doi.org/10.1371/journal.pmed.0040296. Cross-sectional checklist: https://www.strobe-statement.org/checklists/.
