# Supplementary material for: Factors associated with loneliness and social capital in a large volunteer sample of adults in England
Source: BMC Glob Public Health. 2026 Oct 2;4:96. doi: 10.1186/s44263-026-00335-1 (PMC13632327; doi:10.1186/s44263-026-00335-1)
Supplement: Supplementary file 6 [file 44263_2026_335_MOESM6_ESM.docx]

**Supplementary material 6: CHERRIES checklist**

**Table S11: Checklist for Reporting Results of Internet E-Surveys (CHERRIES)**

Reporting is based on the final revised manuscript and the supplied INTERACT survey instrument. Where an operational feature cannot be established from these study records, this is stated explicitly rather than inferred.

| **CHERRIES domain** | **Reporting item** | **INTERACT reporting / location** |
| --- | --- | --- |
| Design | Describe survey design | Cross-sectional secondary analysis of the Measuring Loneliness in England (INTERACT) online survey; non-probability volunteer sample of adults in England. Final manuscript: Methods, opening paragraph. |
| IRB approval | Mention whether the study was approved by an institutional review board | Ethical approval was granted by the NHS Research Ethics Committee (#21IC6950) and Imperial College London Research Ethics Committee (ICREC #305483). The manuscript states that the study conformed to the principles of the Helsinki Declaration. Final manuscript: Declarations. |
| Informed consent | Describe the informed-consent process | All participants provided informed electronic consent before participation. Supplementary material 1, Q1–Q2 provides the study introduction, Participant Information Sheet link and explicit consent question. |
| Data protection / privacy | Describe measures used to protect personal information | The survey introduction states that responses would be anonymous. The final manuscript states that individual-level data cannot be publicly shared and that de-identified data may be made available only subject to institutional approvals and data-protection requirements. Supplementary material 1, Q1; final manuscript: Availability of data and materials. |
| Development and pre-testing | Describe development and testing of the questionnaire | The present manuscript uses established loneliness and social-capital measures and provides complete item wording and scoring in Supplementary material 2. Further questionnaire-development or pre-testing procedures are not independently reported in the final manuscript or supplied survey instrument. |
| Open versus closed survey | State whether the survey was open or closed | The study is described as a non-probability volunteer online survey recruited through NHS primary care networks, voluntary-sector organisations and NIHR Be Part of Research. No login, password or registration requirement is documented in the final manuscript or supplied survey instrument; the survey's technical access configuration therefore cannot be established from these records. |
| Contact mode | Indicate how potential participants were contacted | Participants were recruited through NHS primary care networks, voluntary-sector organisations and the NIHR Be Part of Research Network, with targeted outreach to underrepresented populations. Final manuscript: Participants and Sampling. |
| Advertising the survey | Describe how/where the survey was announced | Recruitment routes are reported in Participants and Sampling. The supplied survey instrument contains the participant-facing introduction, but the exact wording and format of all recruitment messages or advertisements are not available in the supplied records. |
| Web survey platform / administration | Describe the Web survey administration | Responses were collected online via Qualtrics between 1 March and 31 July 2023. Final manuscript: Study Design and Data Source. |
| Context | Describe the context in which the survey was presented | The opening survey text identifies Imperial College London, describes a study of social isolation and loneliness in the community setting, states an estimated completion time of under 10 minutes, and links to the Participant Information Sheet and support resources. Supplementary material 1, Q1. |
| Mandatory / voluntary participation | State whether participation was voluntary | Electronic consent was required before participation. The supplied instrument provides Yes/No consent options. The study is described as a volunteer sample. Supplementary material 1, Q2; final manuscript: Methods and Declarations. |
| Incentives | State whether incentives were offered | Not reported in the final manuscript or supplied survey instrument. No assumption is made about whether incentives were or were not offered. |
| Time/date | State the dates of data collection | Responses used in this analysis were collected between 1 March and 31 July 2023. Final manuscript: Study Design and Data Source. |
| Randomisation of items/questionnaires | Describe any randomisation of items or questionnaires | The supplied survey export shows a defined question order and conditional display logic. No randomisation procedure is reported in the final manuscript or supplied instrument. |
| Adaptive questioning | Describe use of adaptive questioning | The supplied instrument documents at least one conditional display rule: the question on children aged ≤16 years is displayed when the respondent reports having children (Q19–Q20). Other deployed branching or adaptive-question settings cannot be established from the supplied records. |
| Number of items | State the number of questionnaire items | The supplied survey instrument is numbered Q1–Q23 and includes a seven-item social-capital matrix plus two additional COVID-19 matrix statements within Q12. The precise number of separately displayed response fields depends on how matrix items and conditional questions are counted; therefore no alternative item count is inferred. |
| Number of screens/pages | State the number of questionnaire screens/pages | The supplied survey export renders as nine document pages, but this is not evidence of the number of screens/pages presented to respondents in the deployed Qualtrics survey. The deployed screen/page count is not established in the supplied records. |
| Completeness check | Describe mandatory items and completeness checking | Electronic consent was required. Other forced-response or client/server-side completeness checks are not documented in the final manuscript or supplied survey instrument. |
| Review step | State whether respondents could review/change answers before submission | Not documented in the final manuscript or supplied survey instrument. |
| Unique site visitor | Report number of unique visitors if available | Not available from the supplied records. |
| View rate | Report unique survey visitors divided by unique site visitors | Not calculable: unique site-visitor and survey-view counts are not available from the supplied records. |
| Participation rate | Report users who agreed to participate divided by unique first-page visitors | Not calculable from the supplied records because the number of unique first-page visitors is not reported. |
| Completion rate | Report users who finished the survey divided by those who agreed to participate | A CHERRIES completion rate cannot be calculated from the supplied records because started-versus-submitted session counts are not reported. Outcome-specific complete-case counts in the manuscript are analytic completeness measures and are not treated as survey completion rates. |
| Cookies used | Describe whether cookies were used | Cookie use and cookie duration are not documented in the final manuscript or supplied survey instrument. |
| IP check | Describe whether IP addresses were used to identify duplicate entries | IP-address collection or IP-based duplicate checking is not documented in the final manuscript or supplied survey instrument; no such procedure is inferred. |
| Log-file analysis | Describe whether log files were analysed to identify multiple entries | No log-file deduplication procedure is reported in the final manuscript or supplied survey instrument. |
| Registration | Describe whether registration was used to prevent multiple entries | No registration mechanism or repeated-entry prevention rule is documented in the final manuscript or supplied survey instrument. |
| Handling of incomplete questionnaires | Describe how incomplete questionnaires were analysed | Primary analyses used available complete cases separately for each outcome. Multiple imputation by chained equations (five imputed datasets; 40 iterations) was used as a sensitivity analysis. Final manuscript: Handling of missing data and Statistical Analysis; Supplementary material 4. |
| Questionnaires submitted with atypical timestamp | State whether unusually short completion times were excluded | The survey introduction estimated completion in under 10 minutes, but no time-based exclusion criterion or distribution of completion times is reported. No time-based exclusion is inferred. |
| Statistical correction | Describe weighting or other adjustment for non-representative samples | No survey weighting, raking or post-stratification was applied. Analyses are explicitly presented as within-sample, sample-conditional estimates and not population-representative estimates. Covariate adjustment and multiple imputation were used for their stated analytic purposes, not to restore representativeness. Final manuscript: Statistical Analysis. |

**Abbreviations:** CHERRIES, Checklist for Reporting Results of Internet E-Surveys; NHS, National Health Service; NIHR, National Institute for Health and Care Research.

**Source:** Eysenbach G. Improving the quality of Web surveys: the Checklist for Reporting Results of Internet E-Surveys (CHERRIES). J Med Internet Res. 2004;6(3):e34.
